# Our future in the Anthropocene biosphere

**DOI:** 10.1007/s13280-021-01544-8

**Published:** 2021-03-14

**Authors:** Carl Folke, Stephen Polasky, Johan Rockström, Victor Galaz, Frances Westley, Michèle Lamont, Marten Scheffer, Henrik Österblom, Stephen R. Carpenter, F. Stuart Chapin, Karen C. Seto, Elke U. Weber, Beatrice I. Crona, Gretchen C. Daily, Partha Dasgupta, Owen Gaffney, Line J. Gordon, Holger Hoff, Simon A. Levin, Jane Lubchenco, Will Steffen, Brian H. Walker

**Affiliations:** 1 Australian National University, Canberra, Australia; 2 Beijer Institute of Ecological Economics, Royal Swedish Academy of Sciences, Stockholm, Sweden; 3 CSIRO, Canberra, Australia; 4 Global Economic Dynamics and the Biosphere Programme (GEDB), Royal Swedish Academy of Sciences, Stockholm, Sweden; 5 Harvard University, Cambridge, MA USA; 6 Oregon State University, Corvallis, OR USA; 7 Potsdam Institute for Climate Impact Research, Potsdam, Germany; 8 Princeton University, Princeton, NJ USA; 9 Stanford University, Stanford, CA USA; 10 Stockholm Resilience Centre, Stockholm University, Stockholm, Sweden; 11 University of Alaska, Fairbanks, AK USA; 12 University of Cambridge, Cambridge, UK; 13 University of Minnesota, St. Paul, MN USA; 14 University of Waterloo, Waterloo, ON Canada; 15 University of Wisconsin, Madison, WI USA; 16 Wageningen University & Research, Wageningen, The Netherlands; 17 Yale University, New Haven, USA

**Keywords:** Anthropocene, Biosphere stewardship, Biodiversity, Climate, Resilience, Social-ecological

## Abstract

The COVID-19 pandemic has exposed an interconnected and tightly coupled globalized world in rapid change. This article sets the scientific stage for understanding and responding to such change for global sustainability and resilient societies. We provide a systemic overview of the current situation where people and nature are dynamically intertwined and embedded in the biosphere, placing shocks and extreme events as part of this dynamic; humanity has become the major force in shaping the future of the Earth system as a whole; and the scale and pace of the human dimension have caused climate change, rapid loss of biodiversity, growing inequalities, and loss of resilience to deal with uncertainty and surprise. Taken together, human actions are challenging the biosphere foundation for a prosperous development of civilizations. The Anthropocene reality—of rising system-wide turbulence—calls for transformative change towards sustainable futures. Emerging technologies, social innovations, broader shifts in cultural repertoires, as well as a diverse portfolio of active stewardship of human actions in support of a resilient biosphere are highlighted as essential parts of such transformations.

## Introduction

Humans are the dominant force of change on the planet, giving rise to a new epoch referred to as the Anthropocene. This new epoch has profound meaning for humanity and one that we are only beginning to fully comprehend. We now know that society needs to be viewed as part of the biosphere, not separate from it. Depending on the collective actions of humanity, future conditions could be either beneficial or hostile for human life and wellbeing in the Anthropocene biosphere. Whether humanity has the collective wisdom to navigate the Anthropocene to sustain a livable biosphere for people and civilizations, as well as for the rest of life with which we share the planet, is the most formidable challenge facing humanity.

This article provides a systemic overview of the Anthropocene biosphere, a biosphere shaped by human actions. It is structured around the core themes of the first Nobel Prize Summit—Our Planet, Our Future, namely climate change and biodiversity loss, inequality and global sustainability, and science, technology, and innovation to enable societal transformations while anticipating and reducing potential harms (Box 1). These interconnected themes are framed in the context of the biosphere and the Earth system foundation for global sustainability, emphasizing that people and nature are deeply intertwined. Scientific evidence makes clear that both climate change and biodiversity loss are symptoms of the great acceleration of human actions into the Anthropocene, rather than independent phenomena, and that they interact, and interact with social, economic, and cultural development. It emphasizes that efficiency through simplification of our global production ecosystem challenges biosphere resilience in times when resilience is needed more than ever, as a critical asset of flexibility and insurance, for navigating rising turbulence, extreme events, and the profound uncertainty of the Anthropocene. This implies that not only will it be critical to curb human-induced climate change but also to enhance the regenerative capacity of the biosphere, and its diversity, to support and sustain societal development, to collaborate with the planet that is our home, and collaborate in a socially just and sustainable manner. This is the focus of the last part of this article on biosphere stewardship for prosperity. We stress that prosperity and wellbeing for present and future generations will require mobilization, innovation, and narratives of societal transformations that connect development to stewardship of human actions as part of our life-supporting biosphere.

BOX 1 The first Nobel Prize Summit - *Our Planet, Our Future*

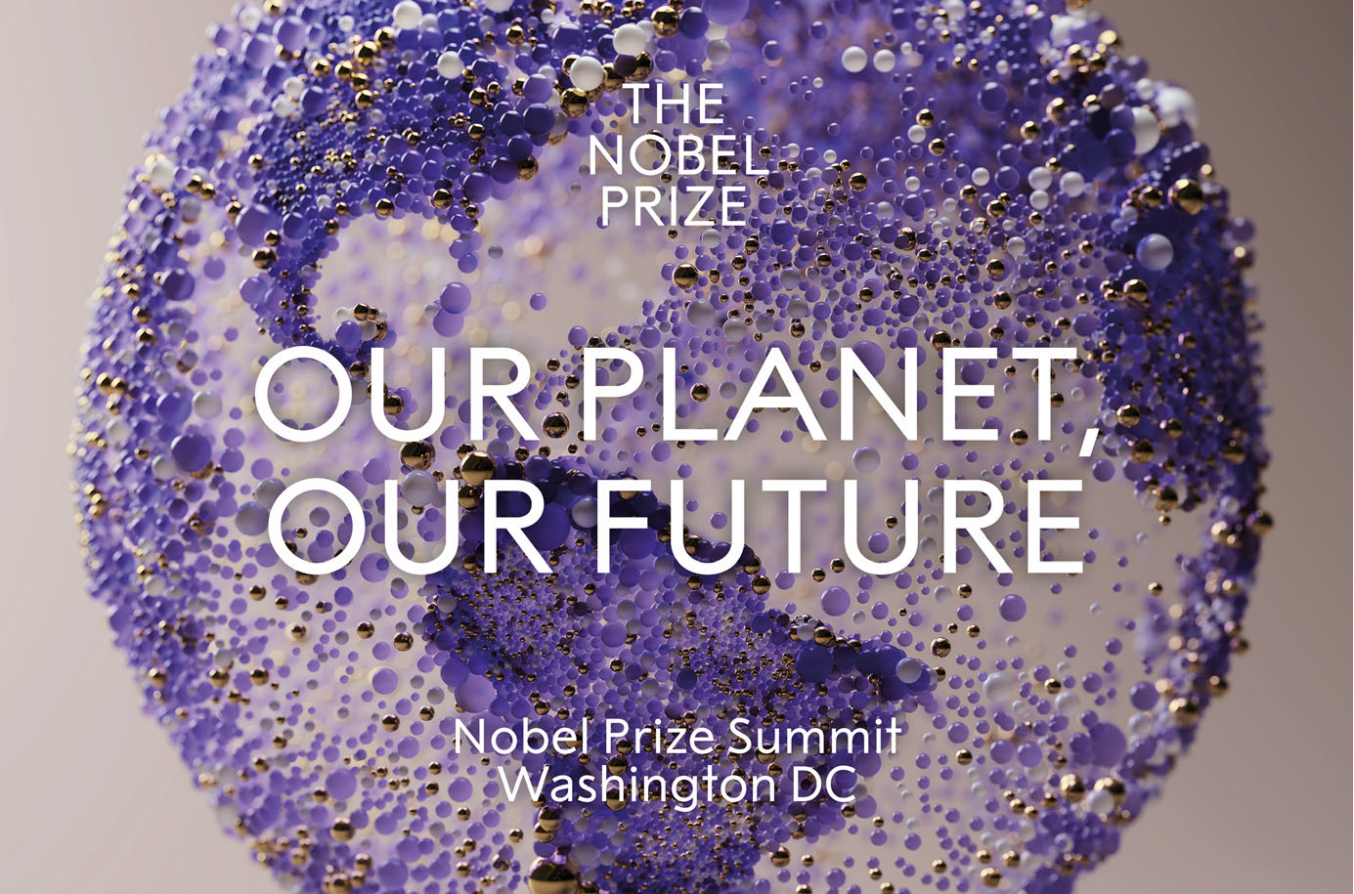
The first Nobel Prize Summit, *Our Planet, Our Future*, is an online convening to discuss the state of the planet at a critical juncture for humanity. The Summit brings together Nobel Laureates and other leading scientists with thought leaders, policy makers, business leaders, and young people to explore solutions to immediate challenges facing our global civilization: mitigate and adapt to the threat posed by climate change and biodiversity loss, reduce inequalities and lift people out of poverty, and made even more urgent due to the economic hardships posed by the pandemic, and harness science, technology, and innovation to enable societal transformations while anticipating and reducing potential harms. The Nobel Prize Summit includes both workshops, publications, and online programmes in forms of webinars, pre-events, and the Nobel Prize Summit days on April 26–28, 2021. The Summit is convened by the Nobel Foundation, in partnership with the U.S. National Academy of Sciences, the Potsdam Institute for Climate Impact Research, and the Stockholm Resilience Centre, Stockholm University/Beijer Institute, Royal Swedish Academy of Sciences. This article is a condensed and updated version of the White Paper “Our future in the Anthropocene biosphere: global sustainability and resilient societies” (Folke et al. 2020) written for the Nobel Prize Summit.

## The biosphere and the earth system foundation

### Embedded in the biosphere

The Universe is immense, estimates suggest at least two trillion galaxies (Conselice et al. 2016). Our galaxy, the Milky Way, holds 100 to 400 billion stars. One of those stars, our sun, has eight planets orbiting it. One of those, planet Earth, has a biosphere, a complex web of life, at its surface. The thickness of this layer is about twenty kilometres (twelve miles). This layer, our biosphere, is the only place where we know life exists. We humans emerged and evolved within the biosphere. Our economies, societies, and cultures are part of it. It is our home.

Across the ocean and the continents, the biosphere integrates all living beings, their diversity, and their relationships. There is a dynamic connection between the living biosphere and the broader Earth system, with the atmosphere, the hydrosphere, the lithosphere, the cryosphere, and the climate system. Life in the biosphere is shaped by the global atmospheric circulation, jet streams, atmospheric rivers, water vapour and precipitation patterns, the spread of ice sheets and glaciers, soil formation, upwelling currents of coastlines, the ocean’s global conveyer belt, the distribution of the ozone layer, movements of the tectonic plates, earthquakes, and volcanic eruptions. Water serves as the bloodstream of the biosphere, and the carbon, nitrogen, and other biogeochemical cycles are essential for all life on Earth (Falkenmark et al. 2019; Steffen et al. 2020). It is the complex adaptive interplay between living organisms, the climate, and broader Earth system processes that has evolved into a resilient biosphere.

The biosphere has existed for about 3.5 billion years. Modern humans (*Homo sapiens*) have effectively been around in the biosphere for some 250 000 years (Mounier and Lahr 2019). Powered by the sun, the biosphere and the Earth system coevolve with human actions as an integral part of this coevolution (Lenton 2016; Jörgensen et al. 2019). Social conditions, health, culture, democracy, power, justice, inequity, matters of security, and even survival are interwoven with the Earth system and its biosphere in a complex interplay of local, regional, and worldwide interactions and dependencies (Folke et al. 2016).

**Fig. 1:**
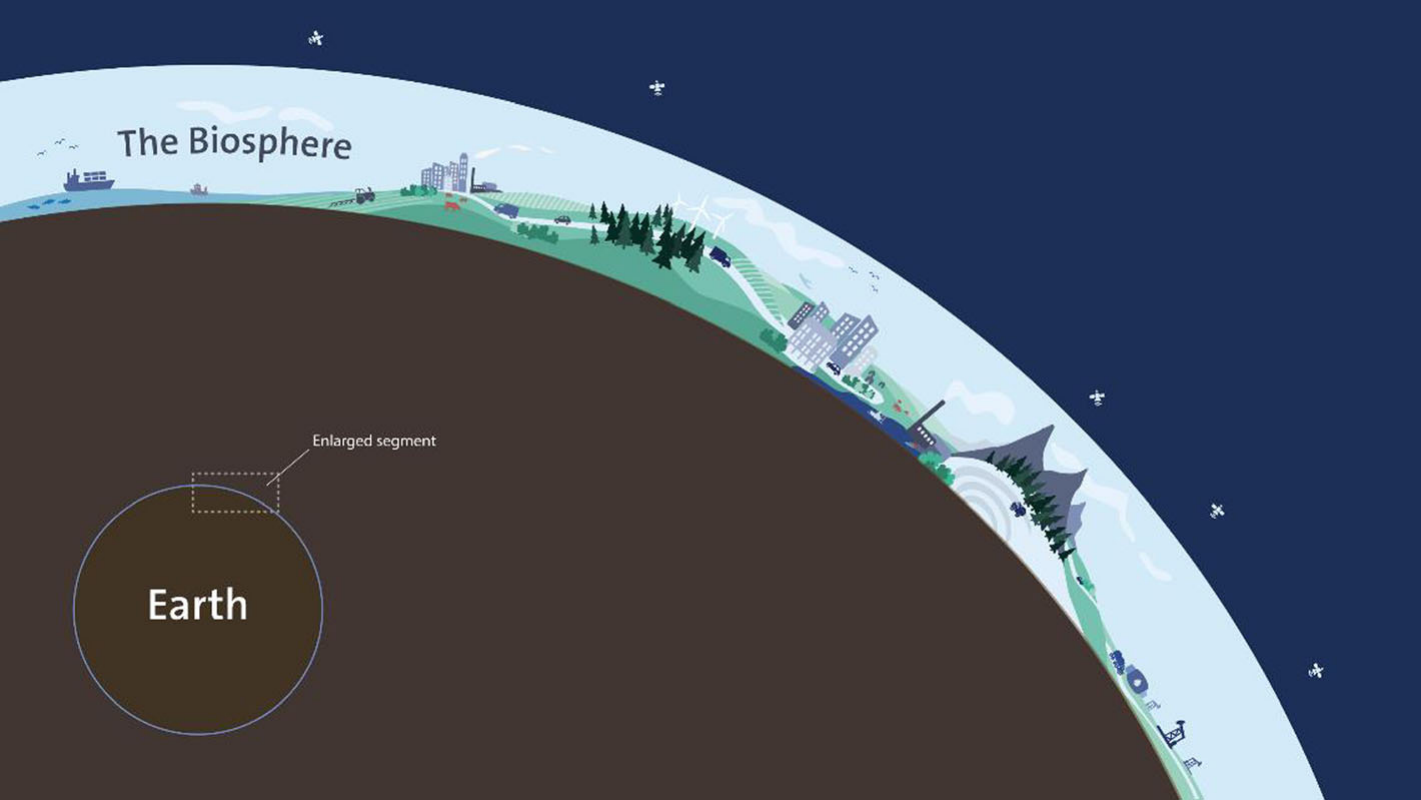
The home of humankind. Our economies, societies, and civilizations are embedded in the Biosphere, the thin layer of life on planet Earth. There is a dynamic interplay between the living biosphere and the broader Earth system, with the atmosphere, the hydrosphere, the lithosphere, the cryosphere, and the climate system. Humans have become a major force in shaping this interplay. Artwork by J. Lokrantz, Azote

Belief systems that view humans and nature as separate entities have emerged with economic development, technological change, and cultural evolution. But the fact that humans are living within and dependent upon a resilient biosphere has and will not change. Existing as embedded within the biosphere means that the environment is not something outside the economy or society, or a driver to be accounted for when preferred, but rather the very foundation that civilizations exist within and rely upon (Fig. 1).

### A dominant force on earth

The human population reached one billion around 1800. It doubled to two billion around 1930, and doubled again to four billion around 1974. The global population is now approaching 8 billion and is expected to stabilize around 9–11 billion towards the end of this century (UN 2019). During the past century, and especially since the 1950s, there has been an amazing acceleration and expansion of human activities into a converging globalized society, supported by the discovery and use of fossil energy and innovations in social organization, technology, and cultural evolution (Ellis 2015; van der Leeuw 2019). Globalization has helped focus attention on human rights, international relations, and agreements leading to collaboration (Keohane et al. 2009; Rogelj et al. 2016; Bain 2019) and, rather remarkably, it appears, at least so far, to have inhibited large-scale conflict between states that have plagued civilizations from time immemorial. Health and material standards of living for many have improved and more people live longer than at any time in history. Boundaries between developed and developing regions have become blurred, and global economic activity is increasingly dispersed across production networks that connect metropolitan areas around the world (Coe et al. 2004; Liu et al. 2015).

Now, there is ample evidence that the cumulative human culture has expanded to such an extent that it has become a significant global force affecting the operation of the Earth system and its biosphere at the planetary level (Steffen et al. 2018). As a reflection of this unprecedented expansion, a new geological epoch—the Anthropocene, the age of mankind—has been proposed in the Geological Time Scale (AWG 2019).

Work on anthropogenic biomes finds that more than 75% of Earth’s ice-free land is directly altered as a result of human activity, with nearly 90% of terrestrial net primary production and 80% of global tree cover under direct human influence (Ellis and Ramankutty 2008). Similarly, in the ocean, no area is unaffected by human influence and a large fraction (41%) is strongly affected by multiple human impacts (Halpern et al. 2008). For example, oxygen-minimum zones for life and oxygen concentrations in both the open ocean and coastal waters have been declining since at least the middle of the twentieth century, as a consequence of rising nutrient loads from human actions coupled with warmer temperatures (Limburg et al. 2020). Just as on land, there has been a blue acceleration in the ocean, with more than 50% of the vast ocean seabed claimed by nations (Jouffray et al. 2020).

The human dominance is further reflected in the weight of the current human population—10 times the weight of all wild mammals. If we add the weight of livestock for human use and consumption to the human weight, only 4% of the weight of mammals on Earth remain wild mammals. The weight of domesticated birds exceeds that of wild birds by about threefold (Bar-On et al. 2018). The human dimension has become a dominant force in shaping evolution of all species on Earth. Through artificial selection and controlled reproduction of crops, livestock, trees, and microorganisms, through varying levels of harvest pressure and selection, through chemicals and pollution altering life-histories of species, and by sculpting the new habitats that blanket the planet, humans, directly and indirectly, determine the constitution of species that succeed and fail (Jörgensen et al. 2019).

Humans are now primarily an urban species, with about 55% of the population living in urban areas. By mid-century, about 7 out of 10 people are expected to live in cities and towns (UN DESA 2018). In terms of urban land area, this is equivalent to building a city the size of New York City every 8 days (Huang et al. 2019). Urbanization leads to more consumption, and the power relations, inequalities, behaviours, and choices of urban dwellers shape landscapes and seascapes and their diversity around the world (Seto et al. 2012a, b). There is growing evidence that urban areas accelerate evolutionary changes for species that play important functional roles in communities and ecosystems (Alberti et al. 2017).

In addition, essential features of the globalized world like physical infrastructure, technological artefacts, novel substances, and associated social and technological networks have been developing extraordinarily fast. The total weight of everything made by humans—from houses and bridges to computers and clothes—is about to exceed the mass of all living things on Earth (Elhacham et al. 2020). The extensive “technosphere” dimension underscores the novelty of the ongoing planetary changes, plays a significant role in shaping global biosphere dynamics, and has already left a deep imprint on the Earth system (Zalasiewicz et al. 2017).

The notion that humanity is external to the biosphere has allowed for models in which technological progress is expected to enable humanity to enjoy ever-growing GDP and thus consumption. This view was comparatively harmless, as long as the biosphere was sufficiently resilient to supply the demands humanity made of it. This is no longer the case, and it has far-reaching implications for contemporary models of economic possibilities that many still work with and draw policy conclusions from (Dasgupta and Ramanathan 2014; Dasgupta 2021).

### The intertwined planet of people and nature

**Fig. 2:**
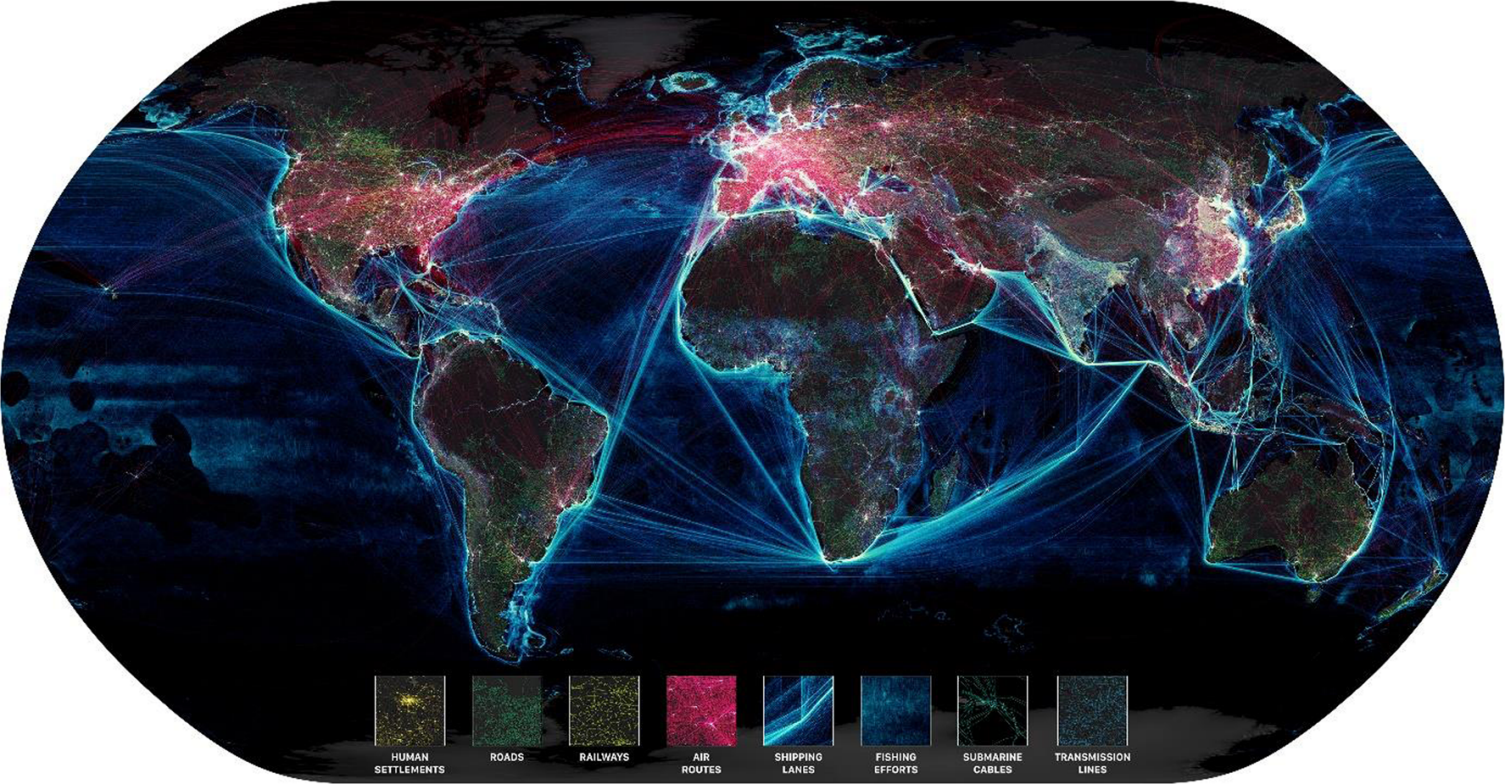
A snapshot of the interconnected globalized world, showing the human influence in terms of settlements, roads, railways, air routes, shipping lanes, fishing efforts, submarine cables, and transmission lines (Credit: Globaïa). Reprinted with permission

The Anthropocene is characterized by a tightly interconnected world operating at high speeds with hyper-efficiency in several dimensions. These dimensions include the globalized food production and distribution system, extensive trade and transport systems, strong connectivity of financial and capital markets, internationalized supply and value chains, widespread movements of people, social innovations, development and exchange of technology, and widespread communication capacities (Helbing 2013) (Fig. 2).

In the Anthropocene biosphere, systems of people and nature are not just linked but intertwined, and intertwined across temporal and spatial scales (Reyers et al. 2018). Local events can escalate into global challenges, and local places are shaped by global dynamics (Adger et al. 2009; Crona et al. 2015, 2016; Liu et al. 2016; Kummu et al. 2020). The tightly coupled human interactions of globalization that allow for the continued flow of information, capital, goods, services, and people, also create global systemic risk (Centeno et al. 2015; Galaz et al. 2017). However, this interplay is not only global between people and societies but co-evolving also with biosphere dynamics shaping the preconditions for human wellbeing and civilizations (Jörgensen et al. 2018; Keys et al. 2019). For example, extreme-weather and geopolitical events, interacting with the dynamics of the food system (Cottrell et al. 2019), can spill over multiple sectors and create synchronous challenges among geographically disconnected areas and rapidly move across countries and regions (Rocha et al. 2018). The rise of antibiotic resistance, the rapid spread of the corona-pandemic, or altered moisture recycling across regions expose the intertwined world. Probabilities and consequences of the changes are not only scale dependent, but also changing over time as a result of human actions, where those actions can either exacerbate or mitigate the likelihood or consequences of a given event.

In the twenty-first century, people and planet are truly interwoven and coevolve, shaping the preconditions for civilizations. Our own future on Earth, as part of the biosphere, is at stake. This new reality has major implications for human wellbeing in the face of climate change, loss of biodiversity, and their interplay, as elaborated in the next section.

## Climate change and loss of biodiversity

Contemporary climate change and biodiversity loss are not isolated phenomena but symptoms of the massive expansion of the human dimension into the Anthropocene. The climate system plays a central role for life on Earth. It sets the boundary for our living conditions. The climate system is integral to all other components of the Earth system, through heat exchange in the ocean, albedo dynamics of the ice sheets, carbon sinks in terrestrial ecosystems, cycles of nutrients and pollutants, and climate forcing through evapotranspiration flows in the hydrological cycle and greenhouse pollutants. Together these interactions in the Earth system interplay with the heat exchange from the sun and the return flow back to space, but also in significant ways with biosphere-climate feedbacks that either mitigate or amplify global warming. These global dynamics interact with regional environmental systems (like ENSO or the monsoon system) that have innate patterns of climate variability and also interact with one another via teleconnections (Steffen et al. 2020). The living organisms of the planet’s ecosystems play a significant role in these complex dynamics (Mace et al. 2014).

Now, human-induced global warming alters the capacity of the ocean, forests, and other ecosystems in sequestering about half of the CO_2_ emissions, as well as storing large amounts of greenhouse gases (GHG) in soils and peatlands (Steffen et al. 2018). Increased emissions of GHG by humans are creating severe climate shocks and extremes already at 1.2° warming compared to pre-industrial levels (WMO 2020). In addition, human homogenization and simplification of landscapes and seascapes cause loss of biosphere resilience, with subsequent erosion of the role of the fabric of nature in generating ecosystem services (Diaz et al. 2018) and serving as insurance to shocks and surprise and to tipping points and regime shifts (Nyström et al. 2019).

### Climate change—stronger and faster than predicted

**Fig. 3:**
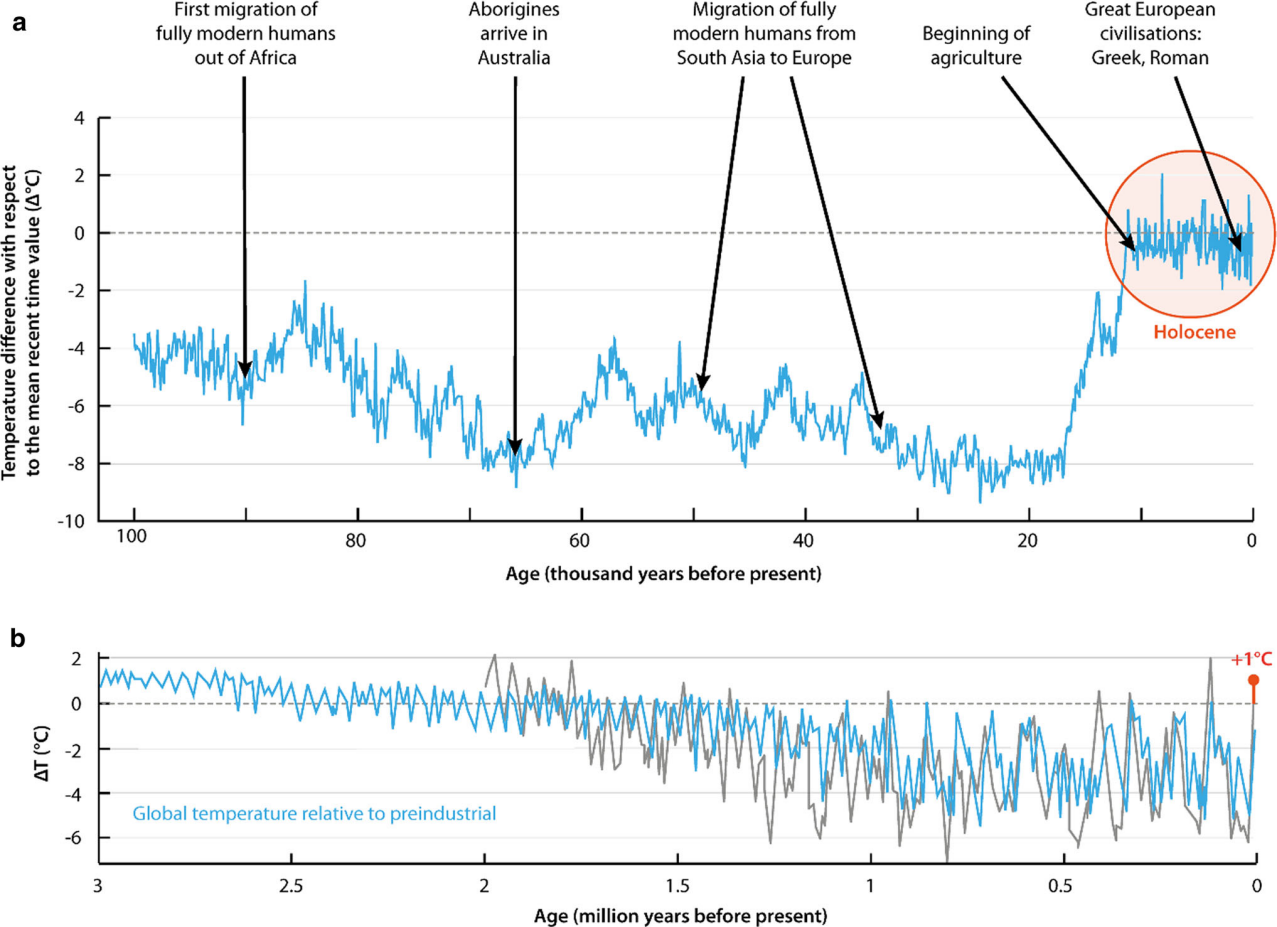
The Holocene epoch and Earth’s resilience. A) Vostok ice-core data, Antarctica, from the last 100 000 years in relation to human migration and civilization. The red circle marks the last 11 000 years of the accommodating Holocene epoch. B) Global temperature the last 3 million years oscillating within + 2 °C and -6 °C relative to pre-industrial temperature (the 0 line). Observations from ice-core and tree ring proxy data in black and modelling results in blue reflecting interactions between the biosphere and the broader Earth system. Evidence suggests that current levels of anthropogenic warming have forced the Earth system out of the Holocene climate conditions into the Anthropocene. There is increasing consensus that pushing the Earth system to more than 2 °C warming compared to pre-industrial levels constitutes unknown terrain for contemporary societies and a threat to civilization (Steffen et al. 2018). Figure 3A by W. Steffen, source and data from Petit et al. (1999) and Oppenheimer (2004). Figure 3B adapted from Willeit et al., *Sci. Adv.* 2019; **5** : eaav7337. © The Authors, some rights reserved; exclusive licensee AAAS. Distributed under a CC BY 4.0 license

Earth has been oscillating between colder and warmer periods over a million years (the entire Pleistocene), but the average mean temperature has never exceeded 2 °C (interglacial) above or 6 °C below (deep ice age) the pre-industrial temperature on Earth (14 °C), reflecting the importance of feedbacks from the living biosphere as part of regulating the temperature dynamics of the Earth (Willeit et al. 2019) (Fig. 3b).

Human-induced global warming is unparalleled. For 98% of the planet’s surface, the warmest period of the past 2000 years occurred in the late twentieth century (Neukom et al. 2019) and has steadily increased into the twenty-first century with the average global temperature for 2015–2020 being the warmest of any equivalent period on record (WMO 2020). Already now at 1.2 °C warming compared to pre-industrial levels, we appear to be moving out of the accommodating Holocene environment that allowed agriculture and complex human societies to develop (Steffen et al. 2018) (Fig. 3a). Already within the coming 50 years, 1 to 3 billion people are projected to experience living conditions that are outside of the climate conditions that have served humanity well over the past 6000 years (Xu et al. 2020).

Currently, some 55% of global anthropogenic emissions causing global warming derive from the production of energy and its use in buildings and transport. The remaining 45% comes from human emissions that arise from the management of land and the production of buildings, vehicles, electronics, clothes, food, packaging, and other goods and materials (Ellen MacArthur Foundation 2019). The food system itself accounts for about 25% of the emissions (Mbow et al. 2019). Human-driven land-use change through agriculture, forestry, and other activities (Lambin and Meyfroidt 2011) causes about 14% of the emissions (Friedlingstein et al. 2020). Cities account for about 70% of CO_2_ emissions from final energy use and the highest emitting 100 urban areas for 18% of the global carbon footprint (Seto et al. 2014; Moran et al. 2018). About 70% of industrial greenhouse gas emissions are linked to 100 fossil-fuel producing companies (Griffin and Hede 2017). Collectively, the top 10 emitting countries account for three quarters of global GHG emissions, while the bottom 100 countries account for only 3.5% (WRI 2020). As a consequence of the pandemic, global fossil CO_2_ emission in 2020 decreased by about 7% compared to 2019 (Friedlingstein et al. 2020).

Climate change impacts are hitting people harder and sooner than envisioned a decade ago (Diffenbaugh 2020). This is especially true for extreme events, like heatwaves, droughts, wildfires, extreme precipitation, floods, storms, and variations in their frequency, magnitude, and duration. The distribution and impacts of extreme events are often region specific (Turco et al. 2018; Yin et al. 2018). For example, Europe has experienced several extreme heat waves since 2000 and the number of heat waves, heavy downpours, and major hurricanes, and the strength of these events, has increased in the United States. The risk for wildfires in Australia has increased by at least 30% since 1900 as a result of anthropogenic climate change (van Oldenborgh et al. 2020). The recent years of repeated wildfires in the western U.S. and Canada have had devastating effects (McWethy et al. 2019). Extreme events have the potential to widen existing inequalities within and between countries and regions (UNDP 2019). In particular, synchronous extremes are risky in a globally connected world and may cause disruptions in global food production (Cottrell et al. 2019; Gaupp et al. 2020). Pandemics, like the COVID-19 outbreak and associated health responses, intersect with climate hazards and are exacerbated by the economic crisis and long-standing socioeconomic and racial disparities, both within countries and across regions (Phillips et al. 2020).

Some of these changes will happen continuously and gradually over time, while others take the form of more sudden and surprising change (Cumming and Peterson 2017). In addition, some are to some extent predictable, others more uncertain and unexpected. An analysis of a large database of social-ecological regime shifts (large shifts in the structure and function of social-ecological systems, transitions that may have substantial impacts on human economies and societies), suggests that in the intertwined world one change may lead to another, or that events can co-occur because they simply share the same driver (Rocha et al. 2018). Large-scale transitions can unfold when a series of linked elements are all close to a tipping point, making it easier for one transition to set off the others like a chain reaction or domino effect (Scheffer et al. 2012; Lenton et al. 2019).

With increased warming, humanity risks departing the glacier-interglacial dynamics of the past 2.6 million years (Burke et al. 2018). If efforts to constrain emissions fail, the global average temperature by 2100 is expected to increase 3–5 °C (IPCC 2014) above pre-industrial levels. Although higher global temperatures have occurred in deep geological time, living in a biosphere with a mean annual global temperature exceeding 2 °C of the pre-industrial average (Fig. 3) is largely unknown terrain for humanity and certainly novel terrain for contemporary society.

### The climate and the biosphere interplay

The relation between climate and the biosphere is being profoundly altered and reshaped by human action. The total amount of carbon stored in terrestrial ecosystems is huge, almost 60 times larger than the current annual emissions of global GHG (CO_2_ equivalents, 2017) by humans, and with the major part, about 70% (1500–2400 Gt C) found in soil (Ciais et al. 2013). The ocean holds a much larger carbon pool, at about 38 000 Gt of carbon (Houghton 2007). Thus far, terrestrial and marine ecosystems have served as important sinks for carbon dioxide and thereby contribute significantly to stabilizing the climate. At current global average temperature, the ocean absorbs about 25% of annual carbon emissions (Gruber et al. 2019) and absorbs over 90% of the additional heat generated from those emissions. Land-based ecosystems like forests, wetlands, and grasslands bind carbon dioxide through growth, and all in all sequester close to 30% of anthropogenic CO_2_ emissions (Global Carbon Project 2019).

The biosphere’s climate stabilization is a critical ecosystem service, or Earth system service, which cannot be taken for granted. Recent research has shown that not only human land-use change but also climate impacts, like extreme events and temperature change, increasingly threaten carbon sinks. For example, the vast fires in Borneo in 1997 released an equivalent of 13–40% of the mean annual global carbon emissions from fossil fuels at that time (Page et al. 2002; Folke et al. 2011). The devastating forest fires of 2019 in Australia, Indonesia, and the Amazon triggered emissions equivalent to almost 40% of the annual global carbon sink on land and in the ocean (www.globalfiredata.org).

**Fig. 4:**
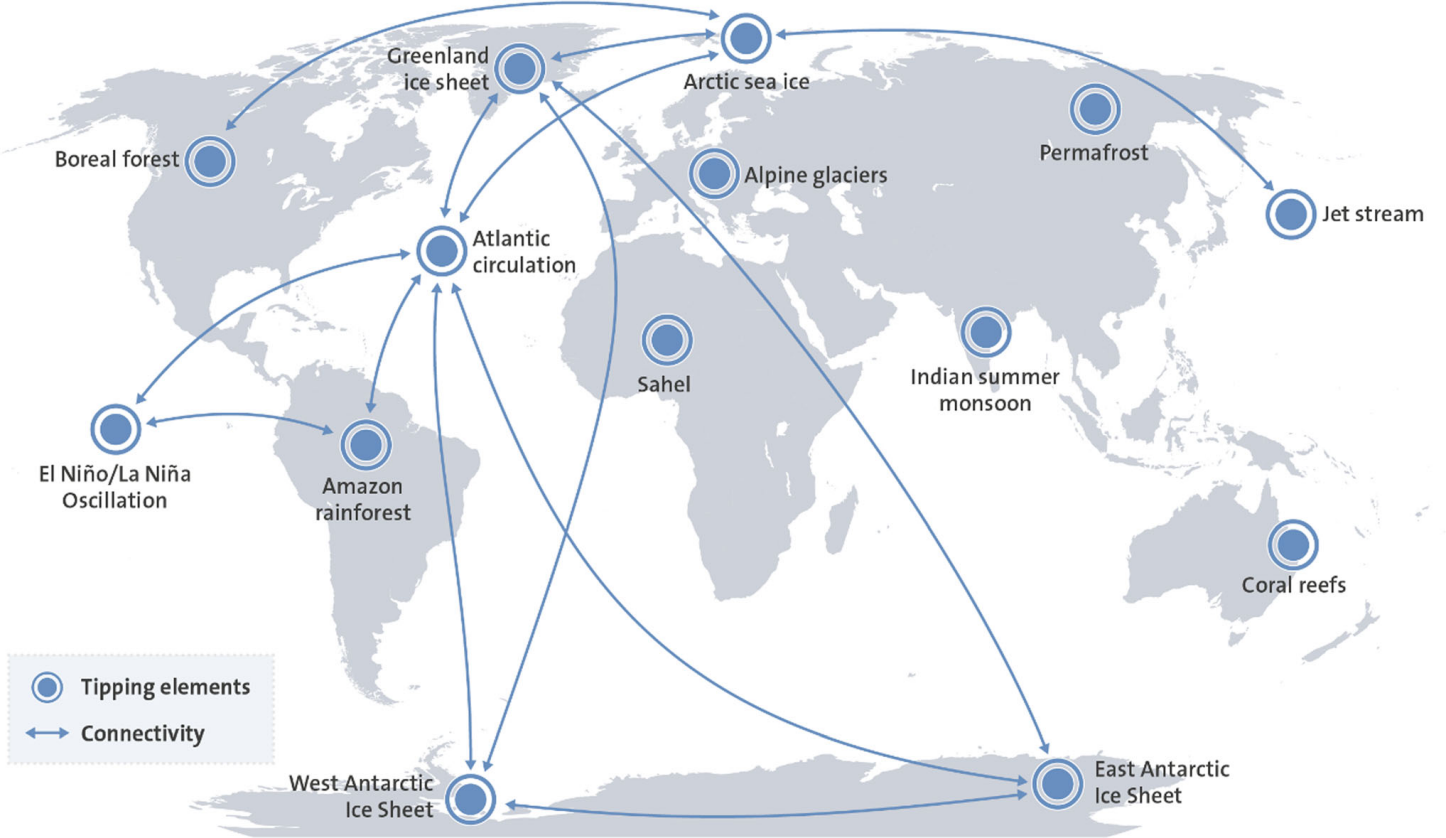
Tipping elements central in regulating the state of the planet, and identified interactions among them that, for humanity, could cause serious cascading effects and even challenge planetary stability (based on Steffen et al. 2018; Lenton et al. 2019). In addition, ocean acidification, deoxygenation, tropical cyclones, ocean heat waves, and sea level rise are challenging human wellbeing (Pörtner et al. 2019)

The Earth system contains several biophysical sub-systems that can exist in multiple states and which contribute to the regulation of the state of the planet as a whole (Steffen et al. 2018). These so-called tipping elements, or sleeping giants (Fig. 4), have been identified as critical in maintaining the planet in favourable Holocene-like conditions. These are now challenged by global warming and human actions, threatening to trigger self-reinforcing feedbacks and cascading effects, which could push the Earth system towards a planetary threshold that, if crossed, could prevent stabilization of the climate at intermediate global warming and cause escalating climate change along a “Hothouse Earth” pathway even as human emissions are reduced (Steffen et al. 2018). Observations find that nine of these known sleeping giants, thought to be reasonably stable, are now undergoing large-scale changes already at current levels of warming, with possible domino effects to come (Lenton et al. 2019).

The significance of the challenge of holding global warming in line with the Paris climate target is obvious. As a matter of fact, the challenge is broader than climate alone. It is about navigating towards a safe-operating space that depends on maintaining a high level of Earth resilience. Incremental tweaking and marginal adjustments will not suffice. Major transformations towards just and sustainable futures are the bright way forward.

### The living biosphere and Earth system dynamics

The interactions and diversity of organisms within and across the planet’s ecosystems play critical roles in the coevolution of the biosphere and the broader Earth system. For example, major biomes like tropical and temperate forests and their biological diversity transpire water vapour that connects distant regions through precipitation (Gleeson et al. 2020a, b). Nearly a fifth of annual average precipitation falling on land is from vegetation-regulated moisture recycling, with several places receiving nearly half their precipitation through this ecosystem service. Such water connections are critical for semi-arid regions reliant on rain-fed agricultural production and for water supply to major cities like Sao Paulo or Rio de Janeiro (Keys et al. 2016). As many as 19 megacities depend for more than a third of their water supply on water vapour from land, a dependence especially relevant during dry years (Keys et al. 2018). In some of the world's largest river basins, precipitation is influenced more strongly by land-use change taking place outside than inside the river basin (Wang-Erlandsson et al. 2018).

The biosphere contains life-supporting ecosystems supplying essential ecosystem services that underpin human wellbeing and socioeconomic development. For example, the biosphere strongly influences the chemical and physical compositions of the atmosphere, and biodiversity contributes through its influence in generating and maintaining soils, controlling pests, pollinating food crops, and participating in biogeochemical cycles (Daily 1997). The ocean’s food webs, continental shelves, and estuaries support the production of seafood, serve as a sink for greenhouse gases, maintain water quality, and hedge against unanticipated ecosystem changes from natural or anthropogenic causes (Worm et al. 2006). These services represent critical life-supporting functions for humanity (Odum 1989; Reyers and Selig 2020) and biological diversity plays fundamental roles in these nature’s contributions to people (Diaz et al. 2018).

### Biodiversity performing vital roles in biosphere resilience

**Fig. 5:**
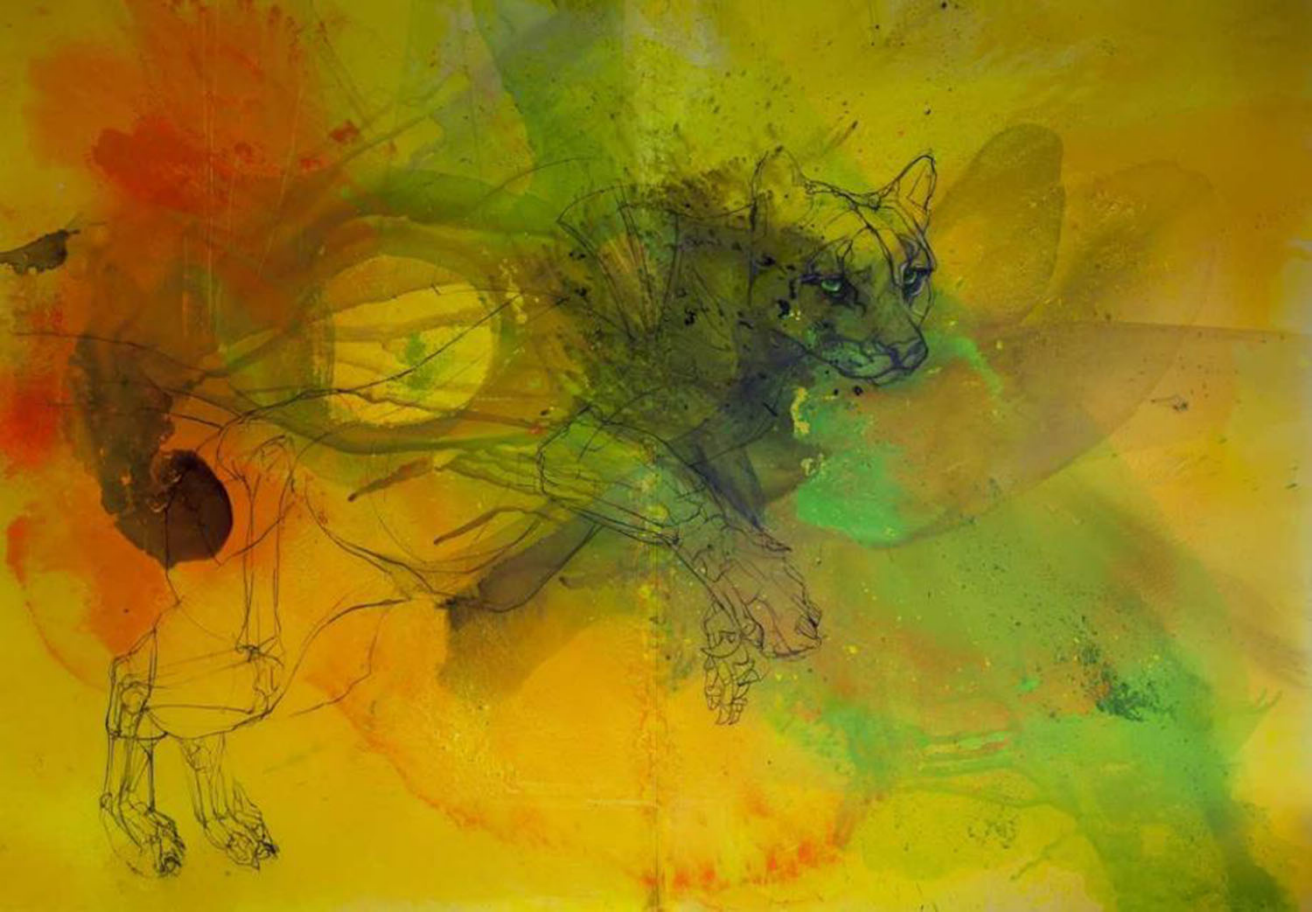
Biodiversity plays significant roles in biosphere resilience. Puma, Kay Pacha 2017, painting, and courtesy of Angela Leible

Organisms do not just exist and compete, they perform critical functions in ecosystem dynamics and in creating and providing social-ecological resilience (Folke et al. 2004; Hooper et al. 2005; Tilman et al. 2014) (Fig. 5). Resilience refers to the capacity of a system to persist with change, to continue to develop with ever changing environments (Reyers et al. 2018).

Biodiversity plays significant roles in buffering shocks and extreme events, and in regime shift dynamics (Folke et al. 2004). The diversity of functional groups and traits of species and populations are essential for ecosystem integrity and the generation of ecosystem services (Peterson et al. 1998; Hughes et al. 2007; Isbell et al. 2017). Variation in responses of species performing the same function is crucial in resilience to shocks or extreme events (Chapin et al. 1997). Such “response diversity”, serves as insurance for the capacity of ecosystems to regenerate, continue to develop after disturbance and support human wellbeing (Elmqvist et al. 2003).

The Amazon rainforest is a prime example. Conserving a diversity of plants species may enable the Amazon forests to adjust to new climate conditions and protect the critical carbon sink function (Sakschewski et al. 2016). Frequent extreme drought events have the potential to destabilize large parts of the Amazon forest especially when subsoil moisture is low (Singh et al. 2020), but the risk of self-amplified forest loss is reduced with increasing heterogeneity in the response of forest patches to reduced rainfall (Zemp et al. 2017). However, continuous deforestation and simultaneous warming are likely to push the forest towards tipping points with wide-ranging implications (Hirota et al. 2011; Staver et al. 2011; Lovejoy and Nobre 2018). Also, with greater climate variability, tree longevity is shortened, thus, influencing carbon accumulation and the role of the Amazon forest as a carbon sink (Brienen et al. 2015). A large-scale shift of the Amazon would cause major impacts on wellbeing far outside the Amazon basin through changes in precipitation and climate regulation, and by linking with other tipping elements in the Earth system (Fig. 4).

Hence, the resilience of multifunctional ecosystems across space and time, and in both aquatic and terrestrial environments, depends on the contributions of many species, and their distribution, redundancy, and richness at multitrophic levels performing critical functions in ecosystems and biosphere dynamics (Mori et al. 2013; Nash et al. 2016; Soliveres et al. 2016; Frei et al. 2020). Biodiversity and a resilient biosphere are a reflection of life continuously being confronted with uncertainty and the unknown. Diversity builds and sustains insurance and keeps systems resilient to changing circumstances (Hendershot et al. 2020).

### Homogenization, hyper-connectivity, and critical transitions

Conversion and degradation of habitats have caused global biodiversity declines and defaunation (human-caused animal loss), with extensive cascading effects in marine, terrestrial, and freshwater ecosystems as a result, and altered ecosystem functions and services (Laliberte et al. 2010; Estes et al. 2011). Over the past 50 years of human acceleration, the capacity of nature to support quality of life has declined in 78% of the 18 categories of nature’s contributions to people considered by the Intergovernmental Science-Policy Platform on Biodiversity and Ecosystem Services (Diaz et al. 2018).

Much of the Earth’s biosphere has been converted into production ecosystems, i.e. ecosystems simplified and homogenized for the production of one or a few harvestable species (Nyström et al. 2019). Urbanization is a force in homogenizing and altering biodiversity in landscapes and seascapes (Seto et al. 2012b), and over the past decade land-use change (Meyfroidt et al. 2018) accounted for nearly a quarter of all anthropogenic greenhouse gas emissions (Arneth et al. 2019).

The increase in homogeneity worldwide denotes the establishment of a global standard food supply, which is relatively species rich at the national level, but species poor globally (Khoury et al. 2014). Globally, local varieties and breeds of domesticated plants and animals are disappearing (Diaz et al. 2018). Land-use intensification homogenizes biodiversity in local assemblages of species worldwide (Newbold et al. 2018) and counteracts a positive association between species richness and dietary quality. It also affects ecosystem services and wellbeing in low- and middle-income countries (Lachat et al. 2018; Vang Rasmussen et al. 2018). In much of the world more than half, up to 90%, of locally adapted varieties of major crop species (e.g. wheat and rice) have been lost due to replacement by single high-yielding varieties (Heal et al. 2004).

The simplification and intensification of production ecosystems and their tight connectivity with international markets have yielded a global production ecosystem that is very efficient in delivering goods to markets, but globally homogeneous, highly interconnected, and characterized by weakened internal feedbacks that mask or dilute the signals of loss of ecosystem resilience to consumers (Nyström et al. 2019; Ortiz et al. 2021). In addition, the global food trade network has over the past 20 years become progressively delocalized as a result of globalization (that is, modularity has been reduced) and as connectivity and homogeneity increase, shocks that were previously contained within a geographical area or a sector are becoming globally contagious and more prevalent (Tamea et al. 2016; Tu et al. 2019; Kummu et al. 2020).

Homogenization reduces resilience, the capacity to live and develop with change and uncertainty, and therby the diversity of ways in which species, people, sectors, and institutions can respond to change as well as their potential to functionally complement each other (Biggs et al. 2012; Grêt-Regamey et al. 2019; Nyström et al. 2019). In addition, homogeneous landscapes lack the diversity of ecosystem types for resilient responses when a single homogeneous landscape patch, such as a production forest or crop, is devastated by pathogens or declines in economic value. In addition, such ecosystem simplification and degradation increase the likelihood of disease emergence, including novel viruses (Myers and Patz 2009). In parallel, people, places, cultures, and economies are increasingly linked across geographical locations and socioeconomic contexts, making people and planet intertwined at all scales.

Evidence suggests that homogenization, simplification, intensification, strong connections, as well as suppression of variance, increase the likelihood of regime shifts, or critical transitions with thresholds and tipping points (Scheffer et al. 2012; Carpenter et al. 2015). These shifts may interact and cascade, thereby causing change at very large scales with severe implications for the wellbeing of human societies (Hughes et al. 2013; Rocha et al. 2018). Comparison of the present extent of biosphere conversion with past global-scale regime shifts suggests that global-scale biosphere regime shift is more than plausible (Barnosky et al. 2012). The biotic hallmark for each earlier biosphere regime shifts was pronounced change in global, regional, and local assemblages of species (Barnosky et al. 2012).

### Planetary boundaries and a safe-operating space for humanity

It is in the self-interest of humanity to avoid pushing ecosystems or the entire Earth system across tipping points. Therefore, a major challenge is to enhance biosphere resilience and work towards stabilizing the Earth system and its biosphere in a state that, hopefully, is safe for humanity to operate within, albeit a warmer state than the Holocene and one with a human-dominated biosphere. Clearly, the climatic system and the biological diversity and functional integrity of the biosphere, as well as their interplay, are foundational for cultivating a resilient Earth system.

**Fig. 6:**
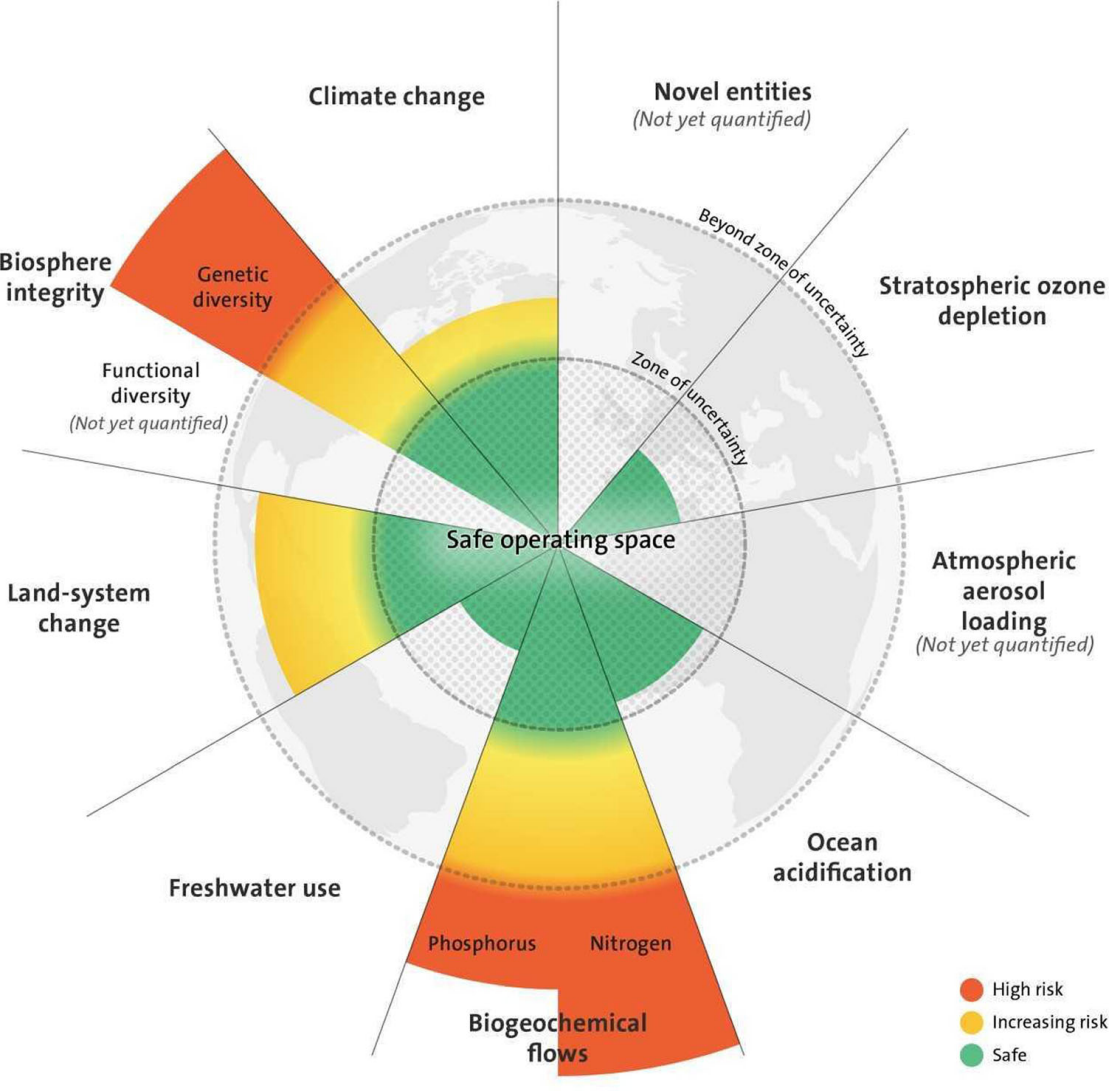
The nine identified planetary boundaries. The green zone is the safe-operating space (below the boundary), yellow represents the zone of uncertainty (increasing risk), and red is the high-risk zone. In these potentially dangerous zones of increasing risk, there are likely continental and global tipping points for some of the boundaries, although not for all them. The planetary boundary itself lies at the inner heavy circle. A proposed boundary does not represent a tipping point or a threshold but is placed upstream of it, that is, well before the risk of crossing a critical threshold. The intent of this buffer between the boundary and a potential threshold in the dangerous zone is to allow society time to react to early warning signs of approaching abrupt or risky change. Processes for which global-level boundaries are not quantified are represented by grey wedges (adapted from Steffen et al. 2015). Reprinted with permission

Climate and biosphere integrity constitute the two fundamental dimensions of the Planetary Boundaries framework, which delineates a Holocene-like state of the Earth system, the state that has enabled civilizations to emerge and flourish (Fig. 6). Four of the nine boundaries, including climate and biodiversity, are estimated to already have been transgressed. The framework provides a natural-science-based observation that human forcing has already, at the planetary scale, rapidly pushed the Earth system away from the Holocene-like conditions and onto an accelerating Anthropocene trajectory (Steffen et al. 2018).

In recent years, there have been several efforts to further investigate and deepen the understanding of planetary boundaries and the safe-operating space for humanity. These include updates on the biodiversity boundary, the freshwater boundary, the biogeochemical flows (Carpenter and Bennett 2011; de Vries et al. 2013; Mace et al. 2014; Newbold et al. 2016; Gleeson et al. 2020b), multiple regime shifts and possible links between regional and planetary tipping points (Anderies et al. 2013; Hughes et al. 2013), regional perspectives on the framework (Häyhä et al. 2016; O’Neill et al. 2018), and creating safe-operating spaces (Scheffer et al. 2015). Attempts to quantify interactions between planetary boundaries suggest that cascades and feedbacks predominantly amplify human impacts on the Earth system and thereby shrink the safe-operating space for human actions in the Anthropocene (Lade et al. 2020).

There are also propositions for integrating the planetary boundaries framework with economic, social, and human dimensions (Raworth 2012; Dearing et al. 2014; Downing et al. 2019) as well as tackling the policy and governance challenges associated with the approach (Biermann et al. 2012; Galaz et al. 2012; Sterner et al. 2019; Pickering and Persson 2020; Engström et al. 2020). The global food system is also placed within the framework of the planetary boundaries (Gordon et al. 2017), like in the EAT-Lancet Commission’s report on healthy diets from sustainable food systems for nearly 10 billion people by 2050 (Willett et al. 2019).

In light of the profound challenges of navigating the future of human societies towards a stabilized Earth state, it becomes clear that modest adjustments on current pathways of societal development are not very likely to guide humanity into sustainable futures (Kates et al. 2012). Stabilizing the Earth system in a safe-operating space will require transformative changes in many dimensions of human actions and relations (Westley et al. 2011; Sachs et al. 2019).

## Inequality and global sustainability

**Fig. 7:**
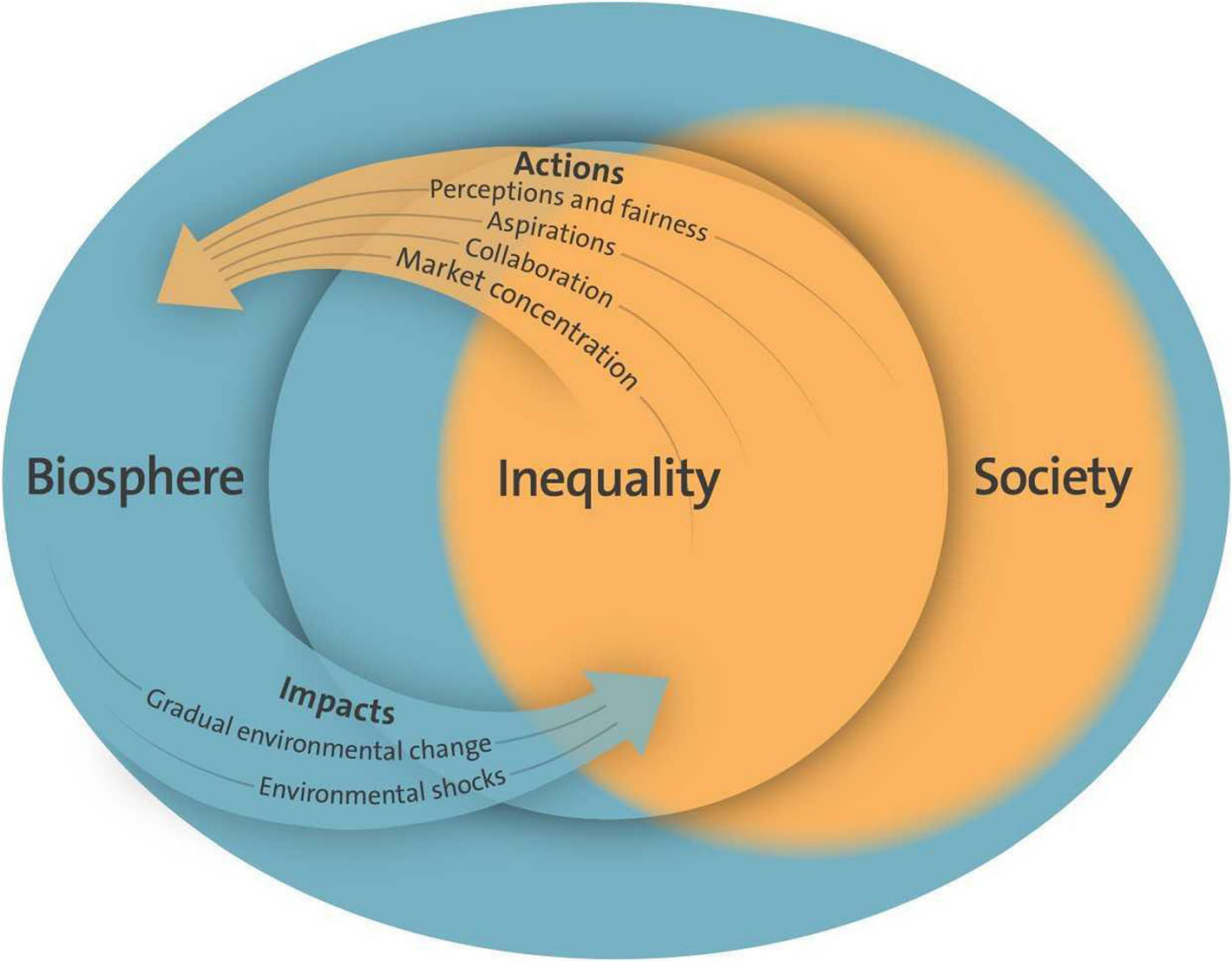
Examples of pathways of interactions between inequality and the biosphere in intertwined systems of people and nature (adapted from Hamann et al. 2018). Reprinted with permission

Inequality describes an unequal distribution of a scarce resource, benefit, or cost and does not necessarily represent a normative statement. Inequity is a more normative term that evokes an unfair or unjust distribution of privileges across society. There are complex interconnections between inequality, the biosphere, and global sustainability (Hamann et al. 2018) (Fig. 7) that go beyond unequal distribution of income or wealth, like distributional, recognitional, and procedural inequities (Leach et al. 2018). Distributional equity refers to how different groups may have access to resources, and how costs, harms, and benefits are shared. Recognitional equity highlights the ongoing struggle for recognition of a diversity of perspectives and groups, e.g. referring to nationality, ethnicity, or gender, whereas procedural equity focuses on how different groups and perspectives are able to engage in and influence decision-making processes and outcomes (Leach et al. 2018). Approaches to sustainability generally include some form of equality, universal prosperity, and poverty alleviation. Global environmental change and unsustainable practices may exacerbate inequalities (Hamann et al. 2018). Greater inequality may lead to weaker economic performance and cause economic instability (Stiglitz 2012). Increasing income inequality may also lead to more societal tension and increase the odds of conflict (Durante et al. 2017).

### Rising inequality

The majority of countries for which adequate data exist have seen rising inequality in income and wealth over the past several decades (Piketty 2014). In the U.S., Europe, and China, the top 10% of the population own 70% of the wealth, while the bottom 50% own only 2%. In the U.S., the share of income going to the top 1% rose from around 11% in 1980 to above 20% in 2016 (World Inequality Report 2018), and the share of wealth of the top 0.1% more than tripled between 1978 and 2012, and is roughly equal to the share of wealth of the bottom 90% (Saez and Zucman 2016). Also, the wealthiest 1% of the world’s population have been responsible for more than twice as much carbon pollution as the poorest half of humanity (Kartha et al. 2020). Seventy-five per cent of the world’s cities have higher levels of income inequalities than two decades ago, and the spatial concentration of low-income unskilled workers in segregated residential areas acts as a poverty trap (UN-Habitat 2016). About 10% of the world population in 2015, or some 740 million people, were living in extreme poverty (World Bank 2019).

Inequality can impact the sense of community, common purpose, and trust (Jachimowicz et al. 2017) and influences successful management of common pool resources in different ways (Baland et al. 2007). Inequality may give rise to perceptions, behaviour, and social norms about status and wealth, and disparities in worth and cultural membership between groups in a society—so-called “recognition gaps” (Lamont 2018).

### Inequalities and the environment

Greater inequality can lead to more rapid environmental degradation, because low incomes lead to low investment in physical capital and education. Such situations often cause excessive pressure and degradation of natural capital leading to declining incomes and further degradation in a downward spiral, a poverty trap (Bowles et al. 2006). Furthermore, interventions that ignore nature and culture can reinforce poverty traps (Lade et al. 2017), and economic and environmental shocks, food insecurity, and climate change may force people back into poverty (lack of resources and capacities to fulfil basic needs) (Kates and Dasgupta 2007; Wood et al. 2018).

Gender, class, caste, and ethnic identities and relationships, and the specific social, economic and political power, roles and responsibilities they entail, shape the choices and decisions open to individuals and households in dealing with the climate and environmental risks they face (Rao et al. 2020). Gender inequality has important reinforcing feedbacks with environmental change (Fortnam et al. 2019) and has, for example, been shown to change with shifts in tropical land use in Indonesia (Maharani et al. 2019) or with changes in levels of direct use of local ecosystem services by households in South Africa (Hamann et al. 2015). Climate change is projected to disproportionally influence disadvantaged groups, especially women, girls, and indigenous communities (Islam and Winkel 2017).

People with less agency and fewer resources at their disposal are more vulnerable to climate change (Althor et al. 2016; Morton 2007) and to environmental shocks and extreme events such as floods and droughts (Hallegatte et al. 2016; Jachimowicz et al. 2017). The COVID-19 pandemic has further exposed the inequality in vulnerability to shocks among communities that lack the financial resources and essentials for a minimum standard of living, feeding off existing inequalities and making them worse (Drefahl et al. 2020; Stiglitz 2020). There is significant concern that climate-driven events exacerbate conflict because they affect economic insecurity which, in itself, has been shown to be a major cause of violent conflict and unrest (Mach et al. 2019; Ide et al. 2020).

Vulnerability to climate change is also due to many low-income countries’ location in low latitudes where further warming pushes these countries ever further away from optimal temperatures for climate-sensitive economic sectors (King and Harrington 2018). Examples include countries with high numbers of vulnerable, poor or marginalized people in climate-sensitive systems like deltas, semi-arid lands, and river basins dependent on glaciers and snowmelt (Conway et al. 2019). Changes to glaciers, snow and ice in mountains will likely influence water availability for over a billion people downstream by mid-century (Pihl et al. 2019). Under future scenarios of land-use and climate change, up to 5 billion people face higher water pollution and insufficient pollination for nutrition, particularly in Africa and South Asia. Hundreds of millions of people face heightened coastal risk across Africa, Eurasia, and the Americas (Chaplin-Kramer et al. 2019).

### Ocean inequity

In the ocean, inequity manifests, for example, in skewed distribution of commercial fish catches, limited political power of small-scale fishers, particularly women and other minority groups, limited engagement of developing nations in high-seas activities and associated decision making, and consolidated interests of global supply chains in a few transnational corporations, with evidence of poor transparency and human rights abuses (Österblom et al. 2019). The results of inequity include a loss of livelihoods and limited financial opportunities, increased vulnerabilities of already marginalized groups, who are facing nutritional and food security challenges, and negative impacts on marine ecosystems (Harper et al. 2013; Hicks et al. 2019).

Coastal communities are sensitive to climate-induced shifts in the distribution and abundance of fish stocks crucial to their livelihoods and nutrition (Blasiak et al. 2017). This accentuated sensitivity is coupled with comparatively low levels of adaptive capacity, as remote coastal communities often have limited access to education, health services and alternative livelihoods, all of which could buffer the projected negative impacts from climate change (Cinner et al. 2018).

As a means to improve fish abundance for coastal communities of low-income nations, there have been suggestions of closing the high seas to fishing through groups of states that commit to a set of international rules. This would not only slow the pace of overfishing, but would also rebuild stocks that migrate into countries’ Exclusive Economic Zones (EEZs), which could reduce inequality by 50% in the distribution of fisheries benefits among the world’s maritime countries (Sumaila et al. 2015; Green and Rudyk 2020).

### Inequities and sustainability

**Fig. 8:**
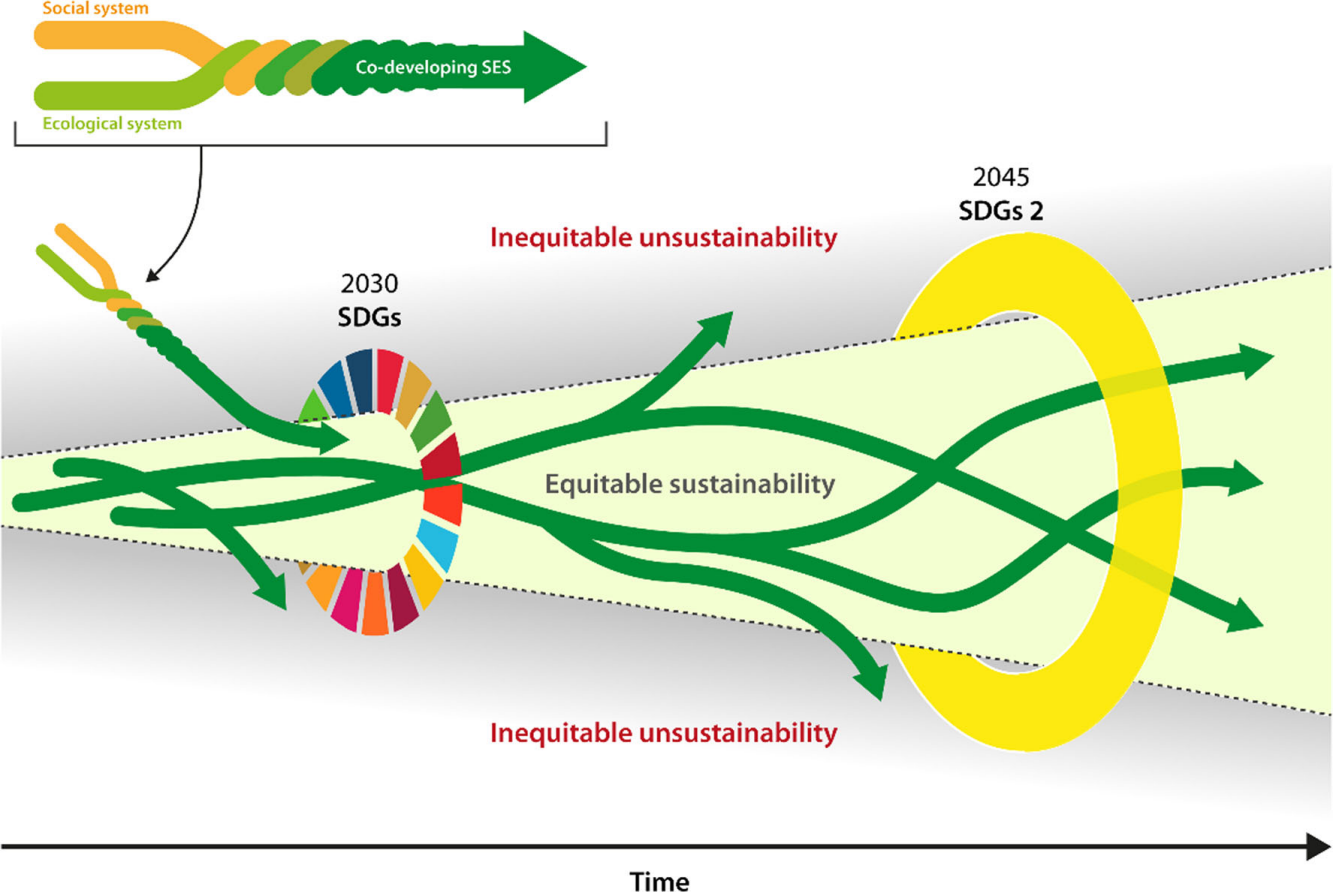
Alternative social-ecological development pathways over time, navigated by efforts like the SDGs and emergent outcomes for equity and sustainability, with an “equitable sustainability space” highlighted (adapted from Leach et al. 2018). Reprinted with permission

Alleviating inequality and poverty is a central objective of the U.N. Sustainable Development Goals agreed to by national governments. Achieving global sustainability is another important set of objectives in the Sustainable Development Goals. The relation between inequality and sustainability is the outcome of this dynamics and not simply of cause and effect, but rather unfolding in different places, as experienced and understood by the people living there. Supporting and enhancing the emergence of capacities for dealing with shocks and surprises as part of strategies for learning and developing with change in the turbulent times of the Anthropocene will be central to confront inequality and advance wellbeing (Biggs et al. 2012; Clark and Harley 2020). Multiple inequities and sustainabilities will require diverse forms of responses, attuned to diverse contexts (Leach et al 2018; Clark and Harley 2020) (Fig. 8) and framed by transformations towards global sustainability as embedded in the biosphere (Westley et al. 2011).

## Societal transformation and technological change

By transformation, we refer to the capacity to create fundamentally new systems of human–environmental interactions and feedbacks when ecological, economic, or social structures make the continuation of the existing system untenable (Folke et al. 2010). It involves multiple elements, including agency, practices, behaviours, incentives, institutions, beliefs, values, and world views and their leverage points at multiple levels (Abson et al. 2017; Moore and Milkoreit 2020). Understanding transformation goes beyond a focus on the triggers, to unravelling the capacities for reducing resilience of an undesired, status quo, system, and nurturing and navigating the emergence of new, desired systems (Elmqvist et al. 2019); to confront path-dependencies, build capacities for new shocks and risks, and shift towards sustainable pathways (Olsson et al. 2017).

Here, we stress that technological change and social innovation in relation to sustainability will need a deeper focus on intertwined social-ecological interactions and feedbacks of the Anthropocene, since that will be necessary to understand and achieve large-scale changes towards global sustainability. We start this section with the role of emerging technologies and social media in this context, followed by findings from social innovation and transformation research and with an emphasis on the significance of narratives of hope for shifting towards sustainable futures.

### Emerging technologies and sustainability

Most likely, technological change such as information technology, artificial intelligence, and synthetic biology will drastically change economies, human relations, social organization, culture and civilization, creating new unknown futures. However, technological change alone will not lead to transformations towards sustainability. It could lead humanity in diverse directions, pleasant and unpleasant ones, and with different social and environmental impacts. For example, rapid advances in sequencing technologies and bioinformatics have enabled exploration of the ocean genome, but the capacity to access and use sequence data is inequitably distributed among countries and companies (Blasiak et al. 2018, 2020). The technological dimension of development has to be deliberately and strategically guided, to contribute to just and sustainable futures and guided how and by whom as a central challenge (Galaz 2014; van der Leeuw 2018).

On the other hand, it is most unlikely that transformations to sustainability will happen without the deployment of technologies that, e.g. help build resilience and development on the ground (Brown 2016), support transformations of current food production and innovation systems (Gordon et al. 2017; Costello et al. 2020), and contribute to a shift towards carbon neutral (or even negative) energy systems (Rockström et al. 2017).

The following categories of new technologies are already having bearing on global sustainability: the diversity of existing and emerging renewable energy technologies, like solar cells, hydrogen energy, wind generators, or geothermal heating; technologies that remove greenhouse gases from the atmosphere; the digital transformation, with Artificial Intelligence (AI), satellite remote sensing, quantum computing, and precision agriculture; synthetic biology, including biotechnology and genetic and molecular engineering, by redesigning and using organisms to solve problems in medicine, manufacturing and agriculture; mechanical engineering, like robotics and also nanotechnology. Their development, as embedded in the larger social-ecological systems, should be connected to and become part of ways forward when designing transformative pathways towards sustainability within planetary boundaries.

As human pressures on the biosphere increase, so does the hope that rapid advances in AI (including automated decision making, data mining, and predictive analytics) in combination with rapid progresses in sensor technology and robotics, will be able to increase society’s capacities to detect, adapt, and respond to climate and environmental change without creating new vulnerabilities (Joppa 2017). Such technologies are applied in a number of research fields related to the environment and climate change, including environmental monitoring, conservation, and “green” urban planning (Hino et al. 2018; Ilieva and McPhearson 2018; Wearn et al. 2019; Reichstein et al. 2019). While nascent in terms of both scale and impact, such technological “niche-innovations” have the potential to rapidly upscale and shape ecosystems and institutions in multiple geographies (Geels et al. 2017). Such innovations have been claimed to be central for a “digital revolution for sustainable development” (Sachs et al. 2019).

Applications of these technologies have effects that span beyond climate and environmental research and monitoring, and more efficient natural resource use. AI-supported recommender systems as an example, influence consumer choices already today (André et al. 2018). Targeted attacks in social media by social bots, applications of computer algorithms that automatically produce content and interact with humans on social media, “trying to emulate and possibly alter their behavior" (Ferrara et al. 2016; Grinberg et al. 2019), also influence conversations in social media about climate and environmental issues and affect institutions for deliberative democracy (Dryzek et al. 2019).

So far, the technological changes to our social systems have not come about with the purpose of promoting global sustainability (van der Leeuw 2019). This remains true of recent and emerging technologies, such as online social media and information technology, causing changes that are increasingly far-reaching, ambiguous, and largely unregulated (Del Vicario et al. 2016). For example, “online social networks are highly dynamic systems that change as a result of numerous feedbacks between people and machines”. Algorithms suggest connections, to which users respond, and the algorithms, trained to optimize user experience, adapt to the responses. “Together, these interactions and processes alter what information people see and how they view the world” (Bergstrom and Bak-Coleman 2019).

Hence, applications of novel technologies stemming from advancements in AI could at best be benevolent and lead to improved stewardship of landscapes, seascapes, water, or climate dynamics, through improved monitoring and interventions, as well as more effective resource use (Chaplin-Kramer et al. 2019). Negative impacts of novel technologies on vulnerable groups (Barocas et al. 2017) are also pertinent since they diffuse rapidly into society, or when used in sectors with clear impacts on the climate, or on land and ocean ecosystems. This issue needs to be taken seriously as technological changes influence decisions with very long-term climatic and biosphere consequences (Cave and Óhéigeartaigh 2019).

### Social media and social change

The participatory nature of social media gives it a central role in shaping individual attitudes, feelings, and behaviours (Williams et al. 2015; Lazer et al. 2018), can underpin large social mobilization and protests (Steinert-Threlkeld et al. 2015), and influence social norms and policy making (Barbier et al. 2018; Stewart et al. 2019). It is well known that dire warnings can lead to disconnect of the audience if it is not accompanied by a feasible perspective for action (Weber 2015). Social media changes our perception of the world, by promoting a sense of crisis and unfairness. This happens as activist groups seek to muster support (Gerbaudo and Treré 2015) and lifestyle movements seek to inspire alternative choices (Haenfler et al. 2012). For instance, social media catalysed the Arab spring among other things by depicting atrocities of the regime (Breuer et al. 2015), and veganism is promoted by social media campaigns highlighting appalling animal welfare issues (Haenfler et al. 2012).

On the worrying side, isolationism stimulated by social-media-boosted discontent may hamper global cooperation needed to curb global warming, biodiversity loss, wealth concentration, and other trends. On the other hand, social media has powered movements such as school strikes, extinction rebellion, voluntary simplicity, bartering, flight shame, the eat-local movement and veganism to promote a steadily rising global awareness of pressing issues that may ultimately shift social norms (Nyborg et al. 2016), trigger reforms towards sustainability (Otto et al. 2020) and perhaps also towards wealth equalization at all institutional levels (Scheffer et al. 2017).

The combination of discontent and self-organization not only promotes rebellion against the old way of doing things, as in street protests, populist votes, radicalization, and terrorism, but also catalyses the search for alternative ways, as in bartering and sharing platforms, or voluntary simplicity and other lifestyle movements (Haenfler et al. 2012; Carpenter et al. 2019).

The rise of social media and technologies such as bots and profiling has been explosive, and the mere rate of change has made it difficult for society to keep pace (Haenfler et al. 2012). Crowd-sourced fact checking may be combined with computer-assisted analyses and judgements from professionals (Hassan et al. 2019), and labelling quality of media sources ranging from internet fora to newspapers and television stations may alert users to the risk of disinformation and heavy political bias (Pennycook and Rand 2019). With time, such approaches together with legislation, best-practice agreements, and individual skills of judging the quality of sources may catch up to control some of the negative side-effects (Walter et al. 2019).

The emerging picture is that social media have become a global catalyst for social change by facilitating shifts on scales ranging from individual attitudes to broad social norms and institutions. It remains unclear, however, whether this new “invisible hand” will move the world on more sustainable and just pathways. Can the global, fast moving capacity for information sharing and knowledge generation through social media help lead us towards a just world where future generations thrive within the limits of our planet’s capacity?

### Social innovation and transformation

Transformations towards sustainability in the Anthropocene cannot be achieved by adaptation alone, and certainly not by incremental change only, but rather that more fundamental systemic transformations will be needed (Hackmann and St. Clair 2012; Kates et al. 2012; O’Brien 2012). Transformation implies fundamentally rewiring the system, its structure, functions, feedbacks, and properties (Reyers et al. 2018). But, despite such changes, there is hope for systemic transformations with dignity, respect and in democratic fashions (Olsson et al. 2017), in contrast to large-scale disruptive or revolutionary societal transformations like those of earlier civilizations (van der Leeuw 2019). It will require trust building, cooperation, collective action, and flexible institutions (Ostrom 2010; Westley et al. 2011).

A characteristic feature of transformations is that change across different system states (trajectories or pathways) is not predetermined but rather emerges through diverse interactions across scales and among diverse actors (Westley et al. 2011). Therefore, the literature on transformations towards sustainability emphasize framing and navigating transformations rather than controlling those. Work on socio-technical sustainability transitions, social-ecological transformations, and social innovation provide insights into these dynamics (Geels et al. 2017; Olsson et al. 2017; Westley et al. 2017).

These literatures have illustrated the importance of connectivity and cross-level interactions for understanding the role of technological and social innovation and transformative systemic change. The work emphasizes the importance of fostering diverse forms of novelty and innovations at the micro-level, supported by the creation of “transformative spaces”, shielded from the forces of dominant system structures. These allow for experimentation with new mental models, ideas, and practices that could help shift societies onto more desirable pathways (Loorbach et al. 2017; Pereira et al. 2018a, b). The examples of the “Seeds of a Good Anthropocene” project reflect ongoing local experiments that, under the right conditions, could accelerate the adoption of pathways to transformative change (Bennett et al. 2016). As multiple demands and stressors degrade the ocean, transformative change in ocean governance seems required, shifting current economic and social systems towards ocean stewardship, e.g. through incorporation of niche innovations within and across economic sectors and stakeholder communities (Brodie Rudolph et al. 2020).

**Fig. 9:**
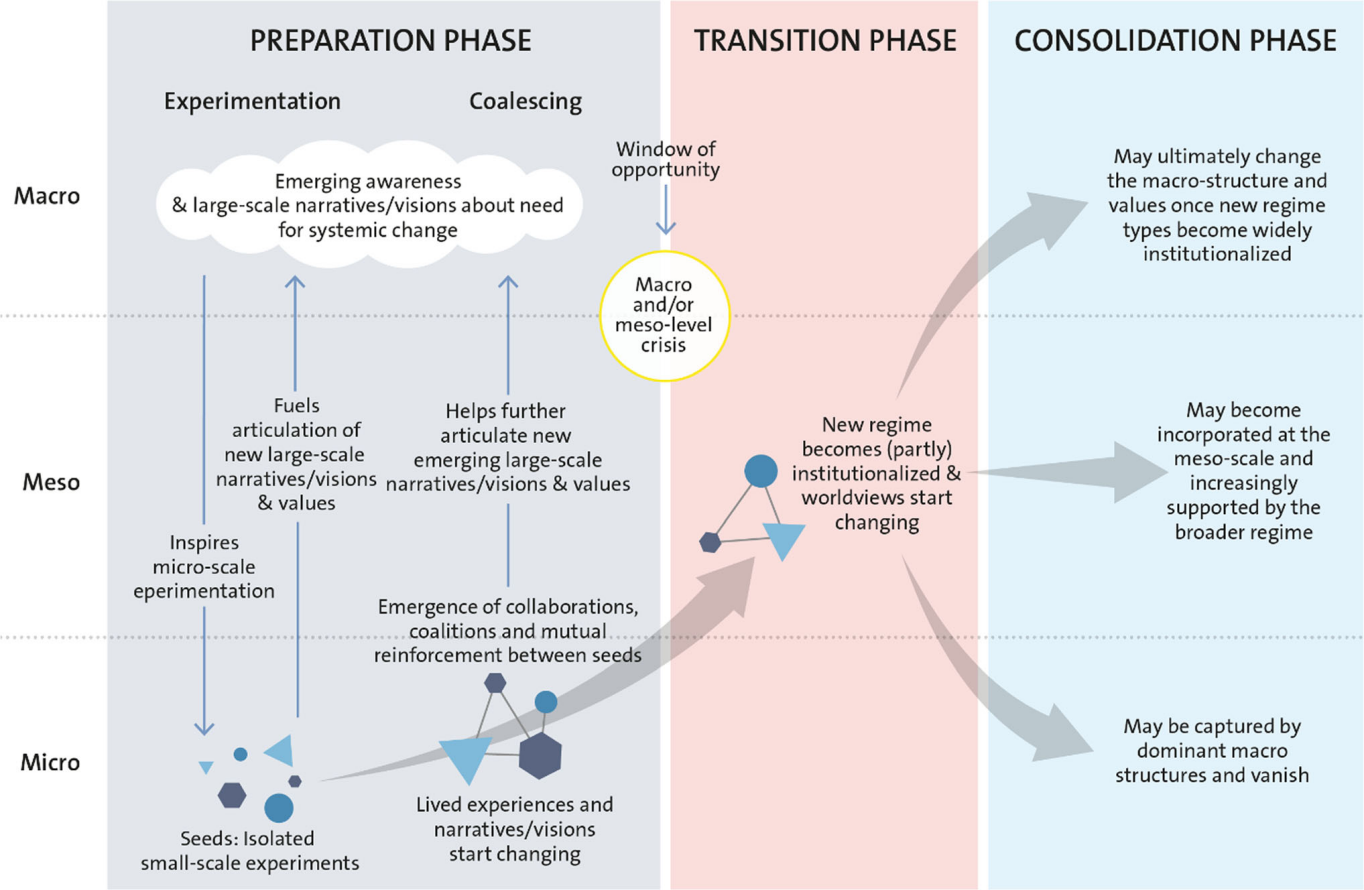
The transformation process. A social innovation, a seed, matures to the extent that the initiative becomes *prepared for change*. And when change happens, when the *window-of-opportunity* unlocks at broader levels of governance, often in relation to a shock or disturbance, the new initiative can be skilfully *navigated through the window and transitioned* into a new development pathway, making it possible to transform the governance system and start *building resilience of the new situation* and taking it to scale (based on Olsson et al. 2004, Geels et al. 2002 and adapted from Pereira et al. 2018b). Reprinted with permission

It has been shown that real-world transformations come about through the alignment of mutually reinforcing processes within and between multiple levels. For example, the alignment of “niche innovations” or “shadow networks’ (which differ radically from the dominant existing system but have been able to gain a foothold in particular market niches or geographical areas) with change at broader levels and scales can create rapid change. Both slow moving trends (e.g., demographics, ideologies, accumulation of GHG) and sudden shocks (e.g. elections, economic crises, pandemics, extreme events) can start to weaken or disturb the existing social-ecological system and create windows-of-opportunity for niche innovations—new practices, governance systems, value orientations—to become rapidly dominant (Olsson et al. 2004, 2006; Chaffin and Gunderson 2016; Geels et al. 2017) (Fig. 9).

Hence, turbulent times may unlock gridlocks and traps and open up space for innovation and novelty (Gunderson and Holling 2002). Crises or anticipated risks can trigger people to experiment with new practices and alternative governance modes and key individuals, often referred to as policy, institutional or moral entrepreneurs, mobilize and combine social networks in new ways, preparing the system for change (Folke et al. 2005; Westley et al. 2013; O’Brien 2015). The preparation phase seems particularly important in building capacity to transform rather than simply returning to the status quo and reinforcing existing power structures following change. Bridging organizations tend to emerge, within or with new institutions, connecting governance levels and spatial and temporal scales (Cash et al. 2006; Hahn et al. 2006; Brondizio et al. 2009; Rathwell and Peterson 2012). In several cases, the broader social contexts provide an enabling environment for such emergence, for example, through various incentive structures or legal frameworks. When a window opens, there is skilful navigation of change past thresholds or tipping points and, thereafter, a focus on building resilience of the transformed system (Gelcich et al. 2010).

In general, the resulting transformation goes beyond the adoption of a new technology or a local social innovation alone. Instead it includes a portfolio of actions like investment in new infrastructures, establishment of new markets, changes in incentives, development of new social preferences, or adjustment of user practices. Furthermore, transformations gain momentum when multiple innovations are linked together, improving the functionality of each and acting in combination to reconfigure systems (Geels et al. 2017; Westley et al. 2017).

Successful social innovations are recognized by their capacity to radically shift broad social institutions (economies, political philosophies, laws, practices, and cultural beliefs) that provide structure to social life. In addition, social innovations seldom unfold in a deterministic manner, but with a kind of punctuated equilibrium, first languishing and then accelerating at times of opportunity or crisis. There is also the need for awareness of the shadow side of all innovation, the consequences of intervention in a complex system (Holling et al. 1998; Ostrom 2007). This is unavoidable but manageable if caught early, but needs attention, particularly in times of rapid change (Westley et al. 2017).

Social innovation is currently underway in many domains linked to climate change, like renewable energy (Geels et al. 2017) or agriculture (Pigford et al. 2018) and highlight the importance of innovations not only in science and technology, but also in institutions, politics, and social goals for sustainability. Substantial attention is also directed towards sustainability of the ocean, where policy makers, industries, and other stakeholders are increasingly engaged in collaboration (Österblom et al. 2017; Brodie Rudolf et al. 2020; UNGC 2020) and innovations (McCauley et al. 2016; Blasiak et al. 2018; Costello et al. 2020), aimed to create new incentives (Lubchenco et al. 2016; Jouffray et al. 2019; Sumaila et al. 2020) for action. However, for these to have transformative impact, shifts in cultural repertoires (schemas, frames, narratives, scripts, and boundaries that actors draw on in social situations) (Lamont et al. 2017) similar to those that accelerated the anti-smoking movement and the LGBTQ movement need to occur (Marshall et al. 2012; Moore et al. 2015; Nyborg et al. 2016).

There are suggestions for social tipping interventions to activate large-scale systemic shifts through, for example, rapidly spreading of technologies, shifts in social norms and behaviors, or structural reorganization of sectors, corporations, and societies (Folke et al. 2019; Otto et al. 2020). There are signs that such shifts are underway in western cultures, a desire for fundamental change towards a more sustainable way of life (Wibeck et al. 2019) aided by social movements such as the youth-led Extinction Rebellion, as well as a strong move to more healthy and sustainable diets (Willet et al. 2019). Again, all these changes unfold as part of cultural evolution, which needs attention as urgently as the decarbonization of our economy (Waring et al. 2015; Creanza et al. 2017; Jörgensen et al. 2019).

### Narratives of action for the future

Social innovation and transformation require an individual and collective attention on the future. There are many documented obstacles to such future focus, from cognitive myopia to present-biased individual and institutional incentives and norms (Weber and Johnson 2016; Weber 2017, 2020). Choice architecture provides tools that reduce status-quo bias and encourage more foresightful decisions in specific circumstances (Yoeli et al. 2017), but rapid and systemic change will require more fundamental shifts in narratives at a collective level (Lubchenco and Gaines 2019).

Narratives are ways of presenting or understanding a situation or series of events that reflects and promotes a particular point of view or set of values. Narratives can serve as meaning‐making devices, provide actors with confidence to act and coordinate action. They are of significance in shaping and anchoring worldviews, identities, and social interactions (van der Leeuw 2020).

Narratives of hope have proven essential for social resilience (Lamont 2019). Social resilience refers to the capacity of individuals, groups, communities, and nations “to secure favourable outcomes (material, symbolic, emotional) under new circumstances and when necessary by new means, even when this entails significant modifications to behaviour or to the social frameworks that structure and give meaning to behaviour” (Hall and Lamont 2012).

Transforming towards sustainable futures will require broadening cultural membership by promoting new narratives that resonate, inspire, and provide hope centred on a plurality of criteria of worth and social inclusion. Here, we are concerned with the challenge of motivating a collective recognition of our interdependence with the biosphere (Schill et al. 2019) and economic and political action based on that recognition.

Collective conceptions of the future have many aspects. They include (1) whether the future is conceived as near or far and is understood in terms of long, medium and short-term rewards; (2) what is likely and possible and how contingent these outcomes are; (3) whether the future will be good or bad; (4) how much agency individuals have on various aspects of their individual and collective future (concerning for instance, politics, societal orientation, personal and professional life; (5) who can influence the collective future (e.g., the role of the state policies and various societal forces in shaping them); (6) whether the future is conceived as a cyclical or as a linear progression; (7) how stable peoples’ conceptions of the future are and how they are influenced by events (terrorist attacks, recessions, pandemics); and (8) whether aspirations are concealed or made public.

Behind these various issues, one finds other basic conceptions about agency (to what extent are individuals master of their fate), the impact of networks (to what extent is fate influenced by peers, family, and others), the impact of social structure (what is the impact of class, race, gender, place of origin) on where we end up, and how much does our environment (segregation, resource availability, environmental conditions) influence our opportunities. Therefore, it is important to remember that, although individuals play essential roles in narratives of hope, such images of the future are seldom creations of individuals alone but shaped by many cultural intermediaries working in the media, in education, in politics, in social movements, and in other institutions.

Cultural scripts represent commonly held assumptions about social interaction, which serve as a kind of interpretive background against which individuals position their own acts and those of others (Lamont et al. 2017). Narratives of hope as cultural scripts are more likely to become widely shared if they offer possible course of action, something that reasonable people can aspire to. Such sharing bolsters people’s sense of agency, the perception that they can have an impact on the world and on their own lives that they can actually achieve what is offered to them (Lamont et al. 2017). In contrast to doomsday or climate-denying narratives, these scripts feed a sense of active agency. Such “fictional expectations”, anchored in narratives that are continually adapted, are at the core of market dynamics confronted with an uncertain future affecting money and credit, investment, innovation, and consumption (Beckert 2016).

Narratives of hope represent ideas about "imagined futures" or alternative ways of visualizing and conceptualizing what has yet to happen and motivate action towards new development pathways (Moore and Milkoreit 2020). As they circulate and become more widely shared, such imagined futures have the potential to foster predictable behaviours, and stimulate the emergence of institutions, investments, new laws, and regulations. Therefore, decisions under uncertainty are not only technical problems easily dealt with by rational calculation but are also a function of the creative elements of decision‐making (Beckert 2016).

There is a rich literature on scenarios for sustainable futures, narratives articulating multiple alternative futures in relation to critical uncertainties, increasingly emphasizing new forms of governance, technology as a bridge between people and the deep reconnection of humanity to the biosphere, and engaging diverse stakeholder in participatory processes as part of the scenario work (Carpenter et al. 2006; Bennett et al. 2016). The implication of inherent unpredictability is that transformations towards sustainable and just futures can realistically be pursued only through strategies that not only attend to the dynamics of the system, but also nurture our collective capacity to guide development pathways in a dynamic, adaptive, and reflexive manner (Clark and Harley 2020; Freeman et al. 2020). Rather than striving to attain some particular future it calls for a system of guided self-organization. It involves anticipating and imagining futures and behaving and acting on those in a manner that does not lead to loss of opportunities to live with changing circumstances, or even better enhances those opportunities, i.e. builds resilience for complexity and change (Berkes et al. 2003).

In order to better understand the complex dynamics of the Anthropocene and uncertain futures, work is now emerging on human behaviour as part of complex adaptive systems (Levin et al. 2013), like anticipatory behaviour (using the future in actual decision processes), or capturing behaviour as both “enculturated” and “enearthed“ and co-evolving with socio-cultural and biophysical contexts (Boyd et al. 2015; Waring et al. 2015; Poli 2017; Merçon et al. 2019; Schill et al. 2019; Schlüter et al. 2019; Haider et al. 2021), illustrating that cultural transmission and evolution can be both continuous and abrupt (Creanza et al. 2017).

Narratives of hope for transformations towards sustainable futures are in demand. Clearly, technological change plays a central role in any societal transformation. Technological change has been instrumental in globalization and will be instrumental for global sustainability. No doubt, the new era of technological breakthroughs will radically change the structure and operation of societies and cultures. But, as has been made clear here, the recipe for sustainable futures also concerns cultural transformations that guide technological change in support of a resilient biosphere; that reconnect development to the biosphere foundation.

## Biosphere stewardship for prosperity

Transformation towards sustainability in the Anthropocene has at least three systemic dimensions. First, it involves a shift in human behaviour away from degrading the life-support foundation of societal development. Second, it requires management and governance of human actions as intertwined and embedded within the biosphere and the broader Earth system. Third, it involves enhancing the capacity to live and develop with change, in the face of complexity and true uncertainty, that is, resilience-building strategies to persist, adapt, or transform. For major pathways for such a transformation are presented in Box 2.

BOX 2 Four major pathwys towards global sustainability

1. Recognize and act on the fact that societal development is embedded in and critically dependent on the biosphere and the broader Earth system for prosperity and wellbeing.
2. Create incentives and design policies that enable societies to collaborate towards just and sustainable futures within planetary boundaries.
3. Transform the current pathways of social, economic, cultural development into stewardship of human actions that enhance the resilience of the biosphere.
4. Make active use of emerging and converging technologies for enabling the societal stewardship transformation.

Biosphere stewardship incorporates economic, social, and cultural dimensions with the purpose of safeguarding the resilience of the biosphere for human wellbeing and fostering the sustainability of a rapidly changing planet. Stewardship is an active shaping of social-ecological change that integrates reducing vulnerability to expected changes, fostering resilience to sustain desirable conditions in the face of the unknown and unexpected, and transforming from undesirable pathways of development when opportunities emerge (Chapin et al. 2010). It involves caring for, looking after, and cultivating a sense of belonging in the biosphere, ranging from people and environments locally to the planet as a whole (Enqvist et al. 2018; Chapin 2020; Plummer et al. 2020).

Such stewardship is not a top-down approach forced on people, nor solely a bottom-up approach. It is a learning-based process with a clear direction, a clear vision, engaging people to collaborate and innovate across levels and scales as integral parts of the systems they govern (Tengö et al. 2014; Clark et al. 2016; Norström et al. 2020).

Here, we focus on biosphere stewardship in relation to climate change, biodiversity, and transformations for sustainable futures.

### From emission reductions alone to biosphere stewardship

Global sustainability involves shifting into a renewable energy-based economy of low waste and greater circularity within a broader value foundation. Market-driven progress combined with technological change certainly plays an important role in dematerialization (Schmidheiny 1992; McAfee 2019) but does not automatically redirect the economy towards sustainable futures. Public awareness, responsible governments, and international collaborations are needed for viable economic developments, acknowledging that people, nations, and the global economy are intertwined with the biosphere and a global force in shaping its dynamics.

Since climate change is not an isolated phenomenon but a consequence of the recent accelerating expansion of human activities on Earth, the needed changes concern social organization and dynamics influencing the emissions of greenhouse gases from burning fossil fuels, technologies, and policies for reducing such emissions, and various approaches for carbon capture and storage. However, to reduce the effects of climate change, it will not be sufficient to remove emissions only. The resilience of the biosphere and the Earth system needs to be regenerated and enhanced (Nyström et al. 2019). This includes governance of critical biosphere processes linked to climate change, such as in agriculture, forestry, and the ocean. In addition, guarding and enhancing biodiversity will help us live with climate change, mitigating climate change by storing and sequestering carbon in ecosystems, and building resilience and adaptive capacity to the inevitable effects of unavoidable climate change (Dasgupta 2021).

**Fig. 10:**
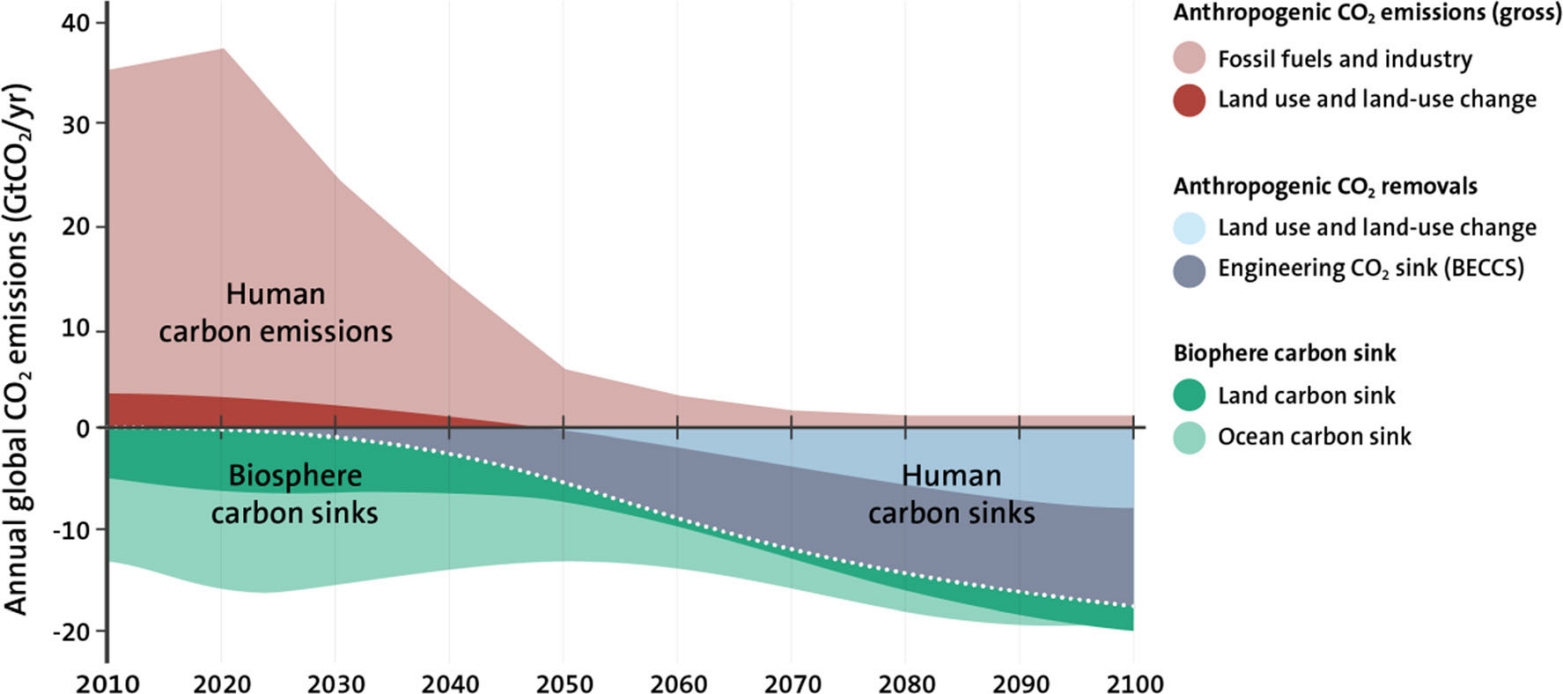
A Roadmap for Rapid Decarbonization—without deep emissions cuts the world takes a high-risk strategy (currently the default strategy) of over-reliance on risky negative emissions technologies in the near future. Avoiding this trap means cutting emissions by half every decade—the Carbon Law trajectory. Meeting the Paris Agreement goals will require bending the global curve of CO_2_ emissions by 2020 and reaching net-zero emissions by 2050. It furthermore depends on rising anthropogenic carbon sinks, by transitioning world agriculture from a major carbon source (red) to become a major carbon sink by the 2nd half of this century, carbon sinks from bioenergy and other forms of carbon capture and storage (BECCS), engineering (grey) and land use (light blue), as well as sustained biosphere carbon sinks, to stabilize global temperatures. Green represents natural carbon sinks, which will shrink as emissions decrease (adapted from Rockström et al. 2017). Reprinted with permission

The global pandemic caused a sharp fall in CO_2_ emissions in 2020 (Le Quéré et al. 2020), while the cumulative emissions continue to rise (Friedlingstein et al. 2020). The fall was not caused by a long-term structural economic shift so it is unlikely to persist without strong government intervention. Political action is emerging from major nations and regions and on net-zero GHG emissions within decades. Shifts towards renewable energy are taking place in diverse sectors. Carbon pricing through taxes, tariffs, tradeable permits, as well as removal of fossil-fuel subsidies and incentives for renewable energy and carbon sequestration (e.g. CCS techniques) are on the table and increasingly implemented. There are substantial material and emission gains to be made from altered consumption patterns, infrastructure changes, and shifts towards a circular economy. Voluntary climate action among some large corporations is emerging (Vandenbergh and Gilligan 2017). There is general agreement that the pace of these promising changes must rapidly increase in order to meet the Paris climate target (Fig. 10).

In addition, active biosphere stewardship of critical tipping elements and carbon sinks, as in forests, agricultural land, savannas, wetlands, and marine ecosystems is crucial to avoid the risk of runaway climate change (Steffen et al. 2018). Such stewardship involves protecting, sustaining, restoring, and enhancing such sinks. The existence of connections between finance actors, capital markets, and the tipping elements of tropical and boreal forests has also gained attention and needs to be acted upon in policy and practice (Galaz et al. 2018).

Furthermore, ecosystem restoration has the potential to sequester large amounts of carbon dioxide from the atmosphere. The amount of carbon dioxide in the atmosphere derived from destroyed and degraded land is roughly equal to the carbon that remains in ecosystems on land (about 450 billion tonnes of carbon) (Erb et al. 2018). The amount of degraded lands in the world is vast, and restoring their productivity, biodiversity, and ecosystem services could help keep global temperature increases within acceptable levels (Lovejoy and Hannah 2018). It has been estimated that nature-based solutions on land (from agriculture to reforestation and afforestation) have the potential to provide over 30% of the emission reductions needed by 2050 to keep global temperature increases to not more than 2 °C (Griscom et al. 2017; Roe et al. 2019).

There is scope for new policies and practices for nature-based solutions (Kremen and Merenlender 2018; Diaz et al. 2018). These solutions will require shifts in governance towards active stewardship of water and ecosystem dynamics and processes across landscapes, precipitation sheds, and seascapes (Österblom et al. 2017; Plummer et al. 2020), reconfiguring nation state governance, empowering the commons through justice, equity and knowledge, and making ownership regenerative by integrating rights with responsibilities (Brodie Rudolph et al. 2020). Also, the so-called “social tipping interventions” towards biosphere stewardship have the potential to activate contagious processes of rapidly spreading technologies, behaviors, social norms, and structural reorganization, where current patterns can be disrupted and lead to fast reduction in anthropogenic greenhouse gas emissions (Otto et al. 2020). The window of opportunity for such shifts may emerge in times of turbulence and social discontent with the status quo (Carpenter et al. 2019). Creating conditions for processes of deliberate democracy may guide such transformative change (Dryzek et al. 2019).

### Resilience and biosphere stewardship

Societal development needs to strengthen biosphere capacity for dealing with extreme events, both climate driven and as a consequence of a tightly coupled and complex globalized world in deep interplay with the rest of the biosphere (Helbing 2013; Reyers et al. 2018). For example, the challenge of policy and practice in satisfying demands for food, water and other critical ecosystem services will most likely be set by the potential consequences of the emergent risk panorama and its consequences, rather than hard upper limits to production per se (Cottrell et al. 2019; Nyström et al. 2019; Xu et al. 2020).

In this sense, a resilience approach to biosphere stewardship becomes significant. Such an approach is very different from those who understand resilience as return to the status quo, to recover to business-as-usual. Resilience in relation to stewardship of complex adaptive systems concerns capacities to live with changing circumstances, slow or abrupt, predictable or surprising. It becomes especially relevant for dealing with the uncertain and unknown and is in stark contrast to strategies that support efficiency and effectiveness for short term gain at the expense of redundancy and diversity. Such strategies may work under relatively stable and predictable conditions but, as stressed here, will create vulnerability in periods of rapid change, during turbulent times, and are ill-suited to confront the unknown (Carpenter et al. 2009; Walker et al. 2009). Financial crises and pandemics serve as real-world examples of such vulnerabilities and make explicit the tension between connectivity and modularity in complex adaptive systems (Levin 1999).

In contrast, intertwined systems of people and nature characterized by resilience will have the capacity, whether through strategies like portfolio management, polycentric institutions, or building trust and nurturing diversity (Costanza et al. 2000; Ostrom 2010; Biggs et al. 2012; Carpenter et al. 2012), to confront turbulent times and the unknown. Policy decisions will no longer be the result of optimization algorithms that presuppose quantifiable uncertainty, but employ decision-making procedures that iteratively identify policy options most robust to present and future shocks under conditions of deep uncertainty (Polasky et al. 2011). Resilience provides capacities for novelty and innovation in times of change, to turn crises into opportunities for not only adapting, but also transforming into sustainable futures (Folke et al. 2016).

The immediate future will require capacities to confront challenges that we know we know little about (Kates and Clark 1996). Given the global connectivity of environmental, social, and economic systems, there is no scale at which resource pooling or trade can be used to hedge against all fluctuations at smaller scales. This begs the question of what types of investments may lead to a generalized capacity to develop with a wide range of potential and unknown events (Polasky et al. 2011). One strategy is to invest in global public goods common to all systems, e.g., education, capacity to learn and collaborate across sectors, multi-scale governance structures that enable systems to better detect changes and nimbly address problems by reconfiguring themselves through transformative change. Such strategies, often referred to as building “general resilience”, easily erode if not actively supported (Biggs et al. 2012; Carpenter et al. 2012; Quinlan et al. 2015). General resilience is critical for keeping options alive to face an uncertain turbulent world (Walker et al. 2009; Elmqvist et al. 2019).

### Collaborating with the biosphere

**Fig. 11:**
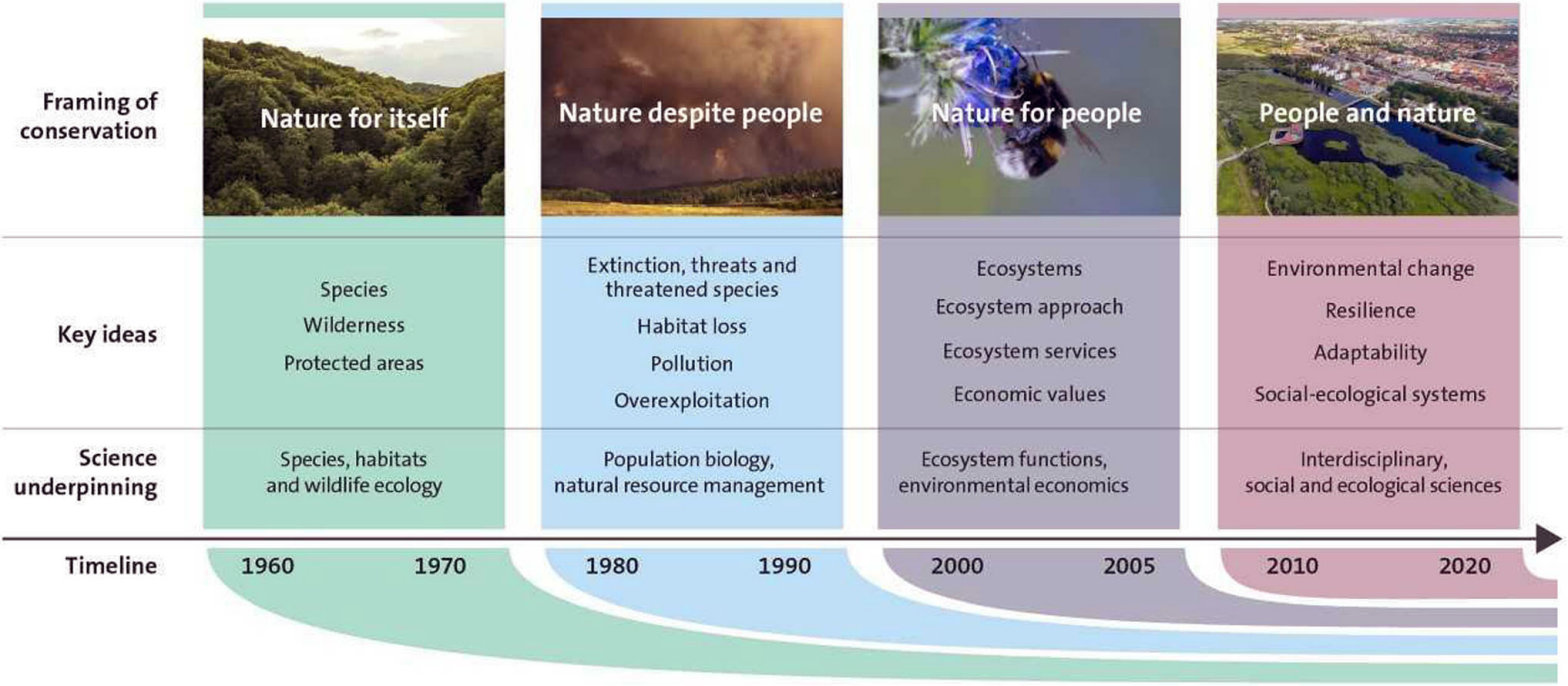
Reconfiguring the human–nature relationship over time (adapted from Mace 2014). Reprinted with permission

Clearly, a shift in perspective and action is needed (Fig. 11) that includes extending management and governance from the focus on producing food, fibre, and timber in simplified ecosystems to rebuilding and strengthening resilience through investing in portfolios of ecosystem services for human wellbeing in diversity-rich social-ecological systems (Reyers et al. 2013; Bennett et al. 2015; Isbell et al. 2017).

Numerous activities protecting, restoring, and enhancing diversity are taking place in this direction ranging from traditional societies, local stewards of wildlife habitats, marine systems, and urban areas, to numerous NGOs, companies and enterprises, and various levels of government, to international collaborations, agreements, and conventions (Barthel et al. 2005; Forbes et al. 2009; Raymond et al. 2010; Andersson et al. 2014; Barrett 2016; Brondizio and Le Tourneau 2016; Österblom et al. 2017; Barbier et al. 2018; Bennett et al. 2018).

Examples include widespread use of marine protected areas from local places to marine spatial planning to proposals for protecting the open ocean, enhancing marine biodiversity, rebuilding fisheries, mitigating climate change, and shifting towards ocean stewardship (Worm et al. 2009; Sumaila et al. 2015; Lubchenco and Grorud-Colvert 2015; Lubchenco et al. 2016; Sala et al. 2016; Gaines et al. 2018; Tittensor et al. 2019; Cinner et al. 2020; Duarte et al. 2020; Brodie Rudolph et al. 2020). The latter is the focus of the High Level Panel for a Sustainable Ocean Economy, with 14 heads of state and more than 250 scientists engaged. They aim to stimulate transformative change for the ocean by committing to sustainably managing 100% of their own waters by 2030 (Stuchtey et al. 2020).

There are major restoration programmes of forests, wetlands, and abandoned and degraded lands and even revival of wildlife and rewilding of nature (Perino et al. 2019). Other efforts include “working-lands conservation” like agroforestry, silvopasture, diversified farming, and ecosystem-based forest management, enhancing livelihoods and food security (Kremen and Merenlender 2018).

The world’s ecosystems can be seen as essential capital assets, if well managed, their lands, waters, and biodiversity yield a flow of vital life-support services (Daily et al. 2009). Investing in natural capital has become a core strategy of agencies and major nations, like China, for wellbeing and sustainability, providing greater resilience to climate change (Guerry et al. 2015; Ouyang et al. 2016). It involves combining science, technology, and partnerships to develop nature-based solutions and enable informed decisions for people and nature to thrive and invest in green growth (Mandle et al. 2019).

There are several examples of adaptive management and adaptive governance systems that have transformed social-ecological dynamics of landscapes and seascapes into biosphere stewardship (Chaffin et al. 2014; Schultz et al. 2015; Walker 2019; Plummer et al. 2020). Stewardship of diversity as a critical feature in resilience building is about reducing vulnerability to change and multiplying the portfolio of options for sustainable development in times of change. Stewardship shifts focus from commodity to redundancy to response diversity for dealing with change (Elmqvist et al. 2003; Grêt-Regamey et al. 2019; Dasgupta 2021).

Clearly, the economic contributions of biodiversity are highly significant as reflected in the many efforts to expose and capture economic values of biodiversity and ecosystem services (Daily et al. 2000; Sukhdev et al. 2010; Kinzig et al. 2011; Costanza et al. 2014; Naeem et al. 2015; Barbier et al. 2018; Dasgupta 2021). Inclusive (or genuine) wealth aims at capturing the aggregate value of natural, human, and social capital assets to provide a comprehensive, long-term foundation for human wellbeing (Dasgupta and Mäler 2000; Polasky et al. 2015). Inclusive wealth provides a basis for designing incentives for more sustainable market transactions (Dasgupta 2014; Clark and Harley 2020).

Also, the role of the cultural context is fundamental (Diaz et al. 2018) and biocultural diversity, and coevolution of people and nature is gaining ground as a means to understand dynamically changing social-ecological relations (Barthel et al. 2013; Merçon et al. 2019; Haider et al. 2019). Broad coalitions among citizens, businesses, nonprofits, and government agencies have the power to transform how we view and act on biosphere stewardship and build Earth resilience. Science has an important new role to play here as honest broker, engaging in evidence-informed action, and coproduction of knowledge in collaboration with practice, policy, and business (Reyers et al. 2015; Wyborn et al. 2019; Norström et al. 2020).

**Fig. 12:**
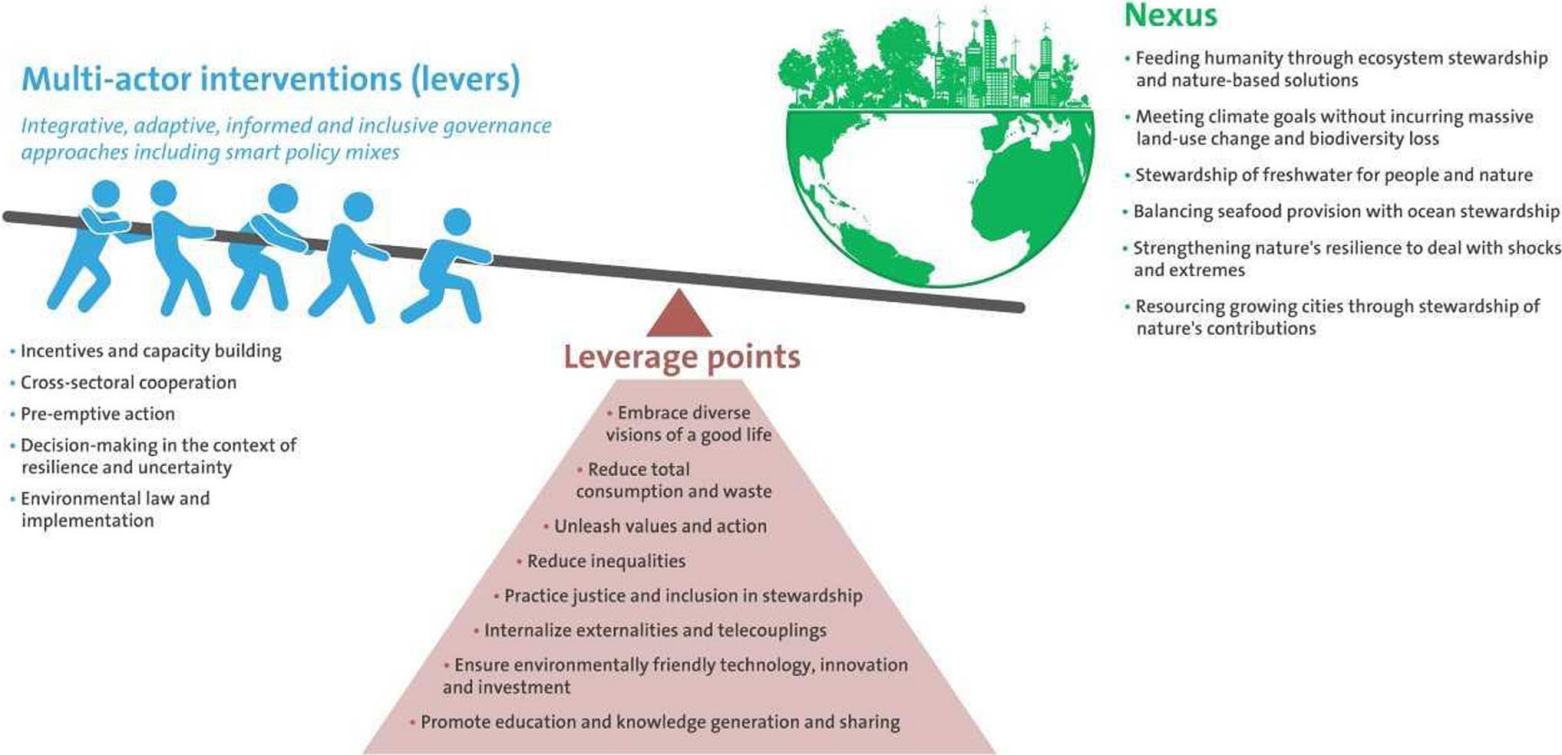
Collaborative implementation of priority interventions (levers) targeting key points of intervention (leverage points representing major indirect drivers) could enable transformative change from current trends towards more sustainable ones. Effectively addressing these levers and leverage points requires innovative governance approaches and organizing the process around nexuses, representing closely interdependent and complementary goals (adapted from Diaz et al. 2018). Reprinted with permission

In this context, work identifying leverage points for anticipated and deliberate transformational change towards sustainability is gaining ground, centred on reconnecting people to nature, restructuring power and institutions, and rethinking how knowledge is created and used in pursuit of sustainability (Abson et al. 2017; Fischer and Riechers 2019). Such actions range from direct engagements between scientists and local communities (Tengö et al. 2014) or through the delivery of scientific knowledge and method into multi-stakeholder arenas, such as boundary or bridging organizations (Cash et al. 2003; Hahn et al. 2006; Crona and Parker 2012) where it can provide a basis for learning and be translated into international negotiations (Biermann and Pattberg 2008; Galaz et al. 2016; Tengö et al. 2017). It includes efforts to accelerate positive transformations by identifying powerful actors, like financial investors or transnational corporations, and articulating key domains with which these actors need to engage in order to enable biosphere stewardship (Österblom et al. 2017; Galaz et al. 2018; Folke et al. 2019; Jouffray et al. 2019). The International science-policy platform for biodiversity and ecosystem services (IPBES), an international body for biodiversity similar to the IPCC for the climate, has proposed key features for enabling transformational change (Fig. 12). These efforts serve an increasingly important space for scientists to engage in, helping hold corporations accountable, stimulating them to take on responsibility for the planet and develop leadership in sustainability. Such science-business engagement will become increasingly important to ensure that companies’ sustainability agendas are framed by science rather than the private sector alone (Österblom et al. 2015; Barbier et al. 2018; Blasiak et al. 2018; Galaz et al. 2018; Folke et al. 2019; Jouffray et al. 2019).

The rapid acceleration of current Earth system changes provides new motivations for action. Climate change is no longer a vague threat to some distant future generation but an environmental, economic, and social disruption that today’s youth, communities, corporations, and governments are increasingly experiencing. This provides both ethical and selfish motivations for individuals and institutions to launch transformative actions that shape their futures rather than simply reacting to crises as they emerge. Shaping the future requires active stewardship for regenerating and strengthening the resilience of the biosphere.

Given the urgency of the situation and the critical challenge of stabilizing the Earth system in Holocene-like conditions, the pace of current actions has to rapidly increase and expand to support a transformation towards active stewardship of human actions in concert with the biosphere foundation. It will require reform of critical social, economic, political, and cultural dimensions (Tallis et al. 2018; Diaz et al. 2018; Barrett et al. 2020).

## Concluding remarks

The success of social organization into civilizations and more recently into a globalized world has been impressive and highly efficient. It has been supported by a resilient biosphere and a hospitable climate. Now, in the Anthropocene, a continuous expansion mimicking the development pathways of the past century is not a viable option for shifting towards sustainable futures.

Humanity is embedded within, intertwined with, and dependent upon the living biosphere. Humanity has become a global force shaping the operation and future of the biosphere and the broader Earth system. Climate change and loss of biodiversity are symptoms of the situation. The accelerating expansion of human activities has eroded biosphere and Earth system resilience and is now challenging human wellbeing, prosperity, and possibly even the persistence of societies and civilizations.

The expansion has led to hyper-connectivity, homogenization, and vulnerability in times of change, in contrast to modularity, redundancy, and resilience to be able to live with changing circumstances. In the Anthropocene, humanity is confronted with turbulent times and with new intertwined dynamics of people and planet where fast and slow change interplay in unexperienced and unpredictable ways. This is becoming the new normal.

Our future on our planet will be determined by our ability to keep global warming well below 2 °C and foster the resilience of the living biosphere. A pervasive thread in science is that building resilient societies, ecosystems, and ultimately the health of the entire Earth system hinges on supporting, restoring and regenerating diversity in intertwined social and ecological dimensions. Diversity builds insurance and keeps systems resilient to changing circumstances. Clearly, nurturing resilience is of great significance in transformations towards sustainability and requires collective action on multiple fronts, action that is already being tested by increasing turbulence incurred by seemingly unrelated shocks.

Equality holds communities together, and enables nations, and regions to evolve along sustainable development trajectories. Inequality, in terms of both social and natural capitals, are on the rise in the world, and need to be addressed as an integral part of our future on Earth.

We are facing a rapid and significant repositioning of sustainability as the lens through which innovation, technology and development is driven and achieved. What only a few years ago was seen as a sacrifice is today creating new purposes and meanings, shaping values and culture, and is increasingly seen as a pathway to novelty, competitiveness and progress.

This is a time when science is needed more than ever. Science provides informed consensus on the facts and trade-offs in times of misinformation and polemics. The planetary challenges that confront humanity need governance that mobilizes the best that science has to offer with shared visions for sustainable futures and political will and competence to implement choices that will sustain humanity and the rest of the living world for the next millennium and beyond.

There is scope for changing the course of history into sustainable pathways. There is urgent need for people, economies, societies and cultures to actively start governing nature’s contributions to wellbeing and building a resilient biosphere for future generations. It is high time to reconnect development to the Earth system foundation through active stewardship of human actions into prosperous futures within planetary boundaries.
